# Estimation of Parent Specific DNA Copy Number in Tumors using High-Density Genotyping Arrays

**DOI:** 10.1371/journal.pcbi.1001060

**Published:** 2011-01-27

**Authors:** Hao Chen, Haipeng Xing, Nancy R. Zhang

**Affiliations:** 1 Department of Statistics, Stanford University, Stanford, California, United States of America; 2 Department of Applied Mathematics and Statistics, SUNY at Stony Brook, Stony Brook, New York, United States of America; 3 Department of Statistics, Stanford University, Stanford, California, United States of America; Accelrys, United States of America

## Abstract

Chromosomal gains and losses comprise an important type of genetic change in tumors, and can now be assayed using microarray hybridization-based experiments. Most current statistical models for DNA copy number estimate total copy number, which do not distinguish between the underlying quantities of the two inherited chromosomes. This latter information, sometimes called *parent specific copy number*, is important for identifying allele-specific amplifications and deletions, for quantifying normal cell contamination, and for giving a more complete molecular portrait of the tumor. We propose a stochastic segmentation model for parent-specific DNA copy number in tumor samples, and give an estimation procedure that is computationally efficient and can be applied to data from the current high density genotyping platforms. The proposed method does not require matched normal samples, and can estimate the unknown genotypes simultaneously with the parent specific copy number. The new method is used to analyze 223 glioblastoma samples from the Cancer Genome Atlas (TCGA) project, giving a more comprehensive summary of the copy number events in these samples. Detailed case studies on these samples reveal the additional insights that can be gained from an allele-specific copy number analysis, such as the quantification of fractional gains and losses, the identification of copy neutral loss of heterozygosity, and the characterization of regions of simultaneous changes of both inherited chromosomes.

## Author Summary

Many genetic diseases are related to copy number aberrations of some regions of the genome. As we know, each chromosome normally has two copies. However, under some circumstances, for some regions, either one or both of the chromosomes change. Genotyping microarray data provides the copy number of the two alleles of polymorphic sites along the chromosomes, which make the inference of the copy number aberrations of the chromosome feasible. One difficulty is that genotyping microarray data cannot provide the haplotype of the two copies of a chromosome. In this paper, we model the copy number along the chromosome as a two-dimensional Markov Chain. Using the observed copy number of both alleles of all the sites, we can determine the parent specific copy number along the chromosome as well as infer the haplotypes of the two copies of the inherited chromosomes in regions where there is allelic imbalance. Simulation results show high sensitivity and specificity of the method. Applying this method to glioblastoma samples from the Cancer Genome Atlas data illustrate the insights gained from allele-specific copy number analysis.

## Introduction

DNA copy number aberration (CNA), defined as gains or losses of specific chromosomal segments, are an important type of genetic change in tumors. Various microarray based experimental platforms [1]–[7] have made possible the fine scale measurement of CNAs. Whereas the earlier platforms such as comparative genome hybridization arrays were designed to measure the total copy number of both inherited chromosomes, other platforms such as high density genotyping microarrays [6]–[8] can measure allele specific DNA quantity. For alleles that represent known variants of genes, it would be of biological interest to know which allele has undergone copy number change [9]. Also, some genetic mechanisms, such as gene conversion, mitotic recombination, and uniparental disomy, cause loss of heterozygosity (LOH) without change in total DNA copy number, and thus can not be detected through conventional analysis methods relying only on total copy number. Even in the case where the total DNA copy number changes, it would be informative to know whether one or both of the inherited parental chromosomes are involved. Thus, to construct a more detailed molecular portrait of tumors, we need to distinguish between the underlying quantities of the two inherited chromosomes, which we call the *parent specific copy numbers*.

This paper addresses the problem of parent specific copy number estimation using allele-specific raw copy number data from high-density genotyping arrays. We will describe the data in more detail in the next section. Here, we clarify the differences between total copy number analysis and parent specific copy number analysis, and review the background of the computational treatment of this problem.

The genome of each somatic human cell normally contains two copies of each of the 22 autosomes, one inherited from each biological parent. At any genome location, one or both of these two chromosomes may gain or lose copies, thus creating a change in total copy number at that location. Microarray experiments for measuring total copy number produce a sequence of continuous valued measurements mapping to ordered locations along the chromosomes. Computational methods can be applied to segment this noisy sequence of measurements into regions of homogeneous copy number [10]–[21], see Lai and Park [22] and Willenbrock and Fridlyand [23] for a review. Since chromosomes are gained and lost in contiguous segments, the true total copy number should be piecewise continuous. This is why change-point models and hidden Markov models have been very useful for total copy number estimation.

**Figure 1:**
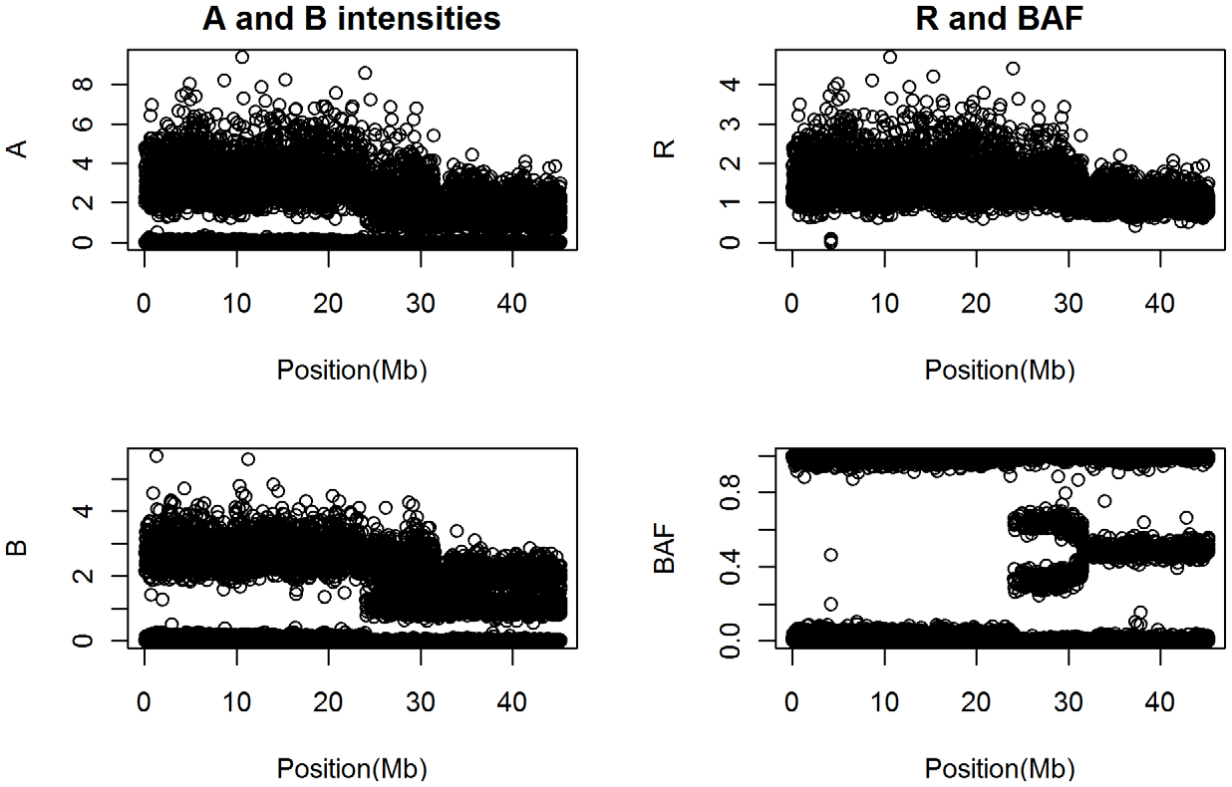
An example data sequence taken from a stretch of a TCGA glioblastoma sample (first 10000 SNPs of TCGA sample 02-0258 chromosome 2) assayed using the Illumina HumanHap 550k SNP array. The left panel shows the A and B allele intensities. The right panel shows the 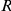 and BAF. All 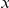-axes are in mega base pairs.

Total copy number estimates do not reveal which (or both) of the two inherited chromosomes have been gained or lost, and if a locus is polymorphic, which (or both) of the alleles have been affected. This information is now available in data produced by high density genotyping platforms, which give, at selected single nucleotide polymorphisms (SNPs), a bivariate measurement quantifying the two alleles which we arbitrarily label 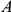 and 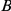, as shown in the left panel of Figure 1. Some platforms output the total raw copy number (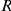), which is the sum of 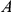 and 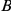, and the B-allele frequency (BAF), which is the percentage of 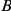 allele raw copy number among the total allele raw copy number, i.e., 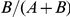. The logR quantifies the total copy number, while the BAF quantifies the imbalance between the two alleles. The right panel of Figure 1 shows 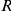, the sum of 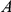 and 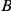 allele intensities, and BAF. Unlike the total copy number, the allele-specific measurements are mixtures that depend on the unknown genotype at each location. For this reason, conventional change-point models can not be applied to allele specific copy number estimation.

This problem can be formulated statistically as follows: The observed 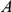 and 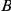 intensities form a bivariate sequence whose underlying distribution undergoes abrupt changes. The distributions at each location are mixtures. Both the change-points, the mixture components, and the cluster memberships at each data point are unknown and must be estimated from the data.

There have been much effort extending existing genotyping and total copy number segmentation procedures to analyze allele-specific data. At the probe level, CNAT [24], CN5 [24], CRMA [25], dChipSNP [26], [27], PLASQ [28], and PICR [29] can be applied to Affymetrix data to produce allele-specific probe-set summaries at each SNP location. However, just as in the estimation of total copy number, the allele-specific intensities for adjacent SNPs should be smoothed to infer the underlying parent-specific copy numbers. LaFramboise et al. [28] first segmented the total copy number using Circular Binary Segmentation [30], and then estimated the parent-specific copy numbers for each segment. This early approach misses copy neutral loss-of -heterozygosity (LOH) events, defined as the simultaneous gain of one chromosome and balanced loss of the other chromosome resulting in loss of heterozygosity but no change in total copy number. Many other existing approaches rely on discrete-state hidden Markov models [27], [31]–[34], which are hidden Markov models assuming a pre-specified finite set of underlying states. For example, PennCNV [32] and QuantiSNP [33] assume that the underlying copy numbers belong to the integer classes 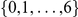, and that the allele-specific copy numbers can be described by “generalized genotypes” AA, AB, BB, A-, B-, AAB, ABB, etc. While these types of models are very useful for detecting germline copy number variants in normal tissue, they do not generalize well to genetically heterogeneous samples. This is because by requiring a fixed set of pre-defined discrete states, they do not account for the heterogeneity of cells within the sample, which produces data with apparently fractional copy number changes rather than the idealized unit-copy changes. This is especially problematic for tumor samples, which are usually heterogeneous mixtures of cells with different genetic profiles. Through titration studies, Staaf et al. [35] showed that methods relying on idealized genotype states lose sensitivity when tumors are diluted with normal cells.

The fractional changes in tumors inspired recent approaches [35], that segment both the logR and BAF simultaneously. Since BAF is a mixture of homozygous and heterozygous SNPs, it cannot be processed using existing segmentation procedures. Current methods solve this problem through a pre-processing step that gets rid of the homozygous SNPs. However, identifying the “homozygous SNPs” is nontrivial when the regions of CNA are unknown, and a segmentation procedure that simultaneously genotype each SNP while inferring the underlying parental copy numbers is desirable, unless a matched normal is available.

In light of these recent developments, we need a systematic stochastic model for parent specific copy number which can accommodate fractional copy number changes. We propose a general two-chromosome hidden Markov model for this problem. The hidden states of the model represent the copy numbers of each of the two inherited chromosomes, and take value in the continuous space of real numbers. Thus, unlike discrete state space HMMs, this model is not limited to idealized unit-copy changes. Computationally efficient fitting algorithms are given that scale well to data obtained from the current high density genotyping arrays. The estimation procedure based on the two chromosome model, which we call Parent-Specific-Copy-Number (PSCN), extends the framework developed in Lai et al. [37] for total copy number analysis.

After segmenting the genome into regions of constant parent-specific copy number, we identify, for each region, whether both or only one of the parental chromosomes have changed copies. We also determine, in regions containing simultaneous gain of one chromosome and loss of the other, whether the changes are balanced. Thus, we classify the regions into six different types of aberrations depending on the status of the two parental chromosomes: gain of both chromosomes (gain/gain), gain of only one chromosome (gain/normal), gain of one chromosome and balanced loss of the other chromosome (balanced gain/loss), gain of one chromosome and unbalanced loss of the other chromosome (unbalanced gain/loss), loss of only one chromosome (normal/loss) and loss of both chromosomes (loss/loss). To our knowledge, this is the most detailed classification available among methods for allele-specific analysis. The PSCN method outputs the copy number for both chromosomes in each segment.

We evaluate the accuracy of the proposed procedure on a series of simulated tumor titration data provided by Staaf et al. [35], as well as a new set of simulation data containing a larger variety of chromosomal aberrations. We then apply the new approach to 223 glioblastoma samples from the Cancer Genome Atlas project [38], and illustrate through case studies some of the insights gained from an analysis of allele-specific data.

## Results

### The Two Chromosome Hidden Markov Model

Let 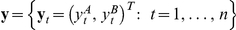 be the allele-specific signals for alleles A and B at 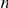 SNPs ordered by their locations in a reference genome. The way of obtaining 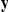 depends on the experimental platform (see “Data Transformation” in Methods). Our goal is to infer the quantities of the parent specific copy numbers, which we denote by 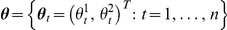. By *parent-specific*, we distinguish between the chromosomes inherited from the two parents, which we treat as exchangeable and do not label as maternal or paternal. Let 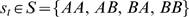 be the configuration at SNP 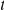 specifying the alleles carried by the inherited chromosomes. Let 
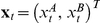
 be the true copy numbers of alleles 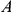 and 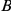 at SNP 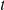. The relationship between 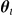, 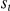, and 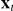 is shown in Table 1.

**Table 1:** Relationship between the inherited allele configuration 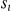 and the true allele specific copy numbers 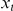.

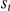	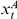	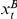
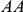	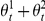	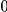
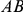	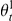	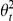
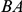	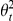	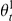
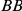	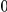	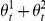

**Figure 2:**
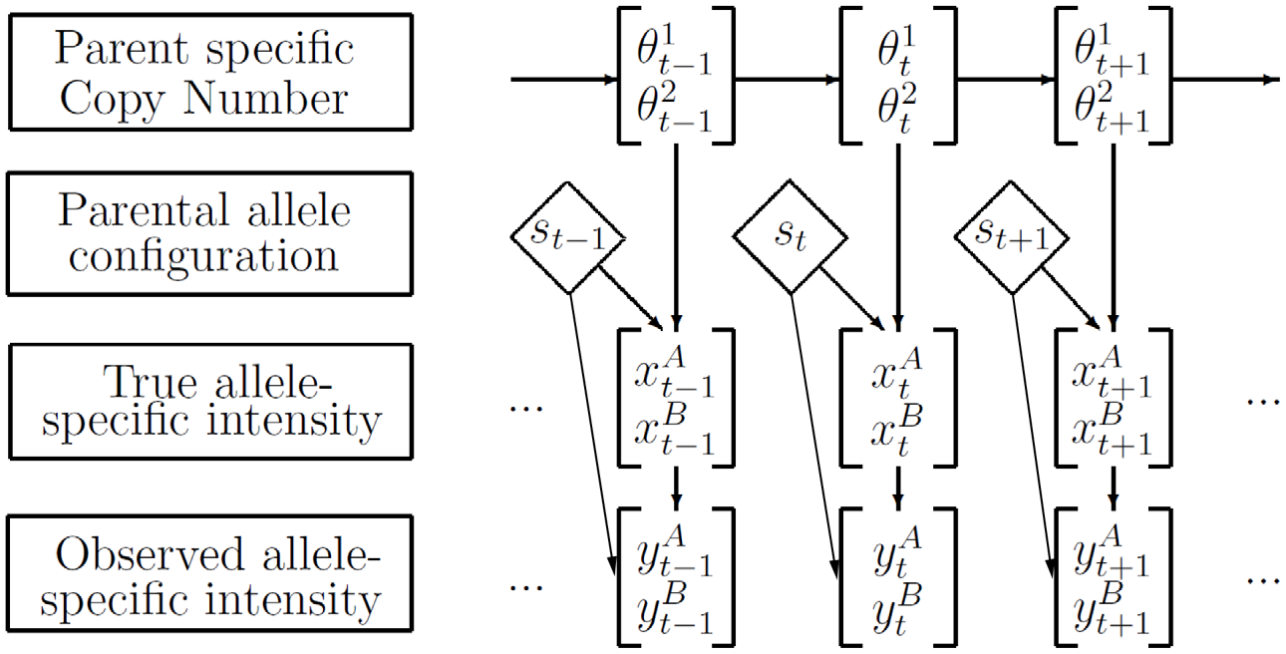
Overview of the stochastic segmentation model. The Markov sequence 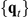 represent the parent-specific copy number, i.e. the underlying copy numbers of the two inherited chromosomes. For each SNP 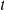, the *allele*-specific copy numbers 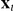 depend on both 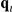 and the inherited allele configuration 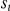. The observed allele-specific signals, 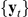, are 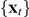 overlayed with Gaussian noise. 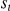 affects 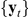 in the way that different type of 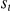 can have different covariance structure for the Gaussion noise.

Note that when a somatic event causes a change in copy number of one or both parental chromosomes at SNP 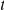, the allele-specific copy numbers 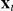 change, but 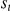 remains fixed. For example, if the inherited genotype is 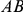, and if 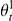 is amplified two-fold, then the true copy number of allele 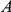 would also be amplified two-fold, but 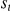 would still be 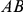. The *observed* allele specific signals 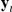 are assumed to be equal to the true allele specific quantities plus an independent measurement error,
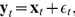
(1)where 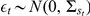 and 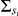 are state specific error covariance matrices. The model that relates 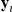 to 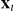, 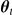 and 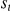 is illustrated in Figure 2.

To model the gains and losses of the two inherited chromosomes, we assume that 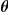 is a Markov jump process with state space 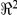. Conceptually, each time 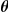 jumps, it can choose between two states: The *normal* state (one copy each of maternal and paternal chromosome), where 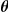 must assume a known baseline value 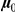, or the *variant* state, where 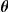 picks a new random value from the bivariate Gaussian 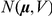. The prior mean 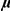 and prior covariance 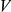, along with the other hyperparameters of the prior, will be estimated by maximum likelihood. To allow the possibility of the copy number changing from a variant state to a different variant state, for example, 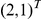 to 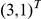, we technically need two identically distributed variant states in our formulation of the Markov chain. Hence we let the states be 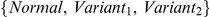. Then, the dynamics of the Markov model can be described by the transition matrix
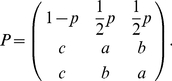
(2)The matrix 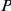 specifies that if 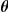 is in the normal state at SNP 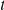, then at SNP 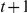, 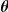 stays in the normal state with probability 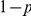, or jumps to a variant state with probability 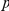. If 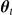 is in a variant state, then at SNP 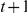, it would stay at the *same* variant state with probability 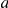, or jump to a *different* variant state with probability 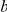, or jump back to the normal state with probability 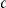. One can verify that this formulation of the Markov chain, with one baseline state and two variant states, allows for a model with a baseline state and generic “variant” states as desired. This model extends the one used for the analysis of total copy number in Lai et al. [37]. This Markov chain has the stationary distribution 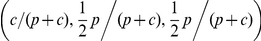. The three-state Markov chain with transition probability matrix 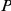 and initialized at the stationary distribution is reversible, which provides substantial simplification for the estimation of 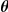. Practically, the reversibility of the Markov model implies that we would obtain the same segmentation going from right to left as we do going from left to right. Biologically, this seems logical, as there is no known directionality of copy number aberration events.

We assume that the inherited allele configurations 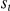 are independent multinomial with prior parameters
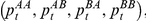
which can be obtained from the genotyping data of a set of normal control samples. Note that 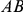 and 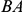 cannot be distinguished in normal samples, so we can set 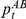 and 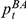 to one-half of the proportion of heterozygotes for SNP 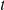. When these figures are not available, we have found that a uniform prior usually works reasonably well. This is because the main purpose of the model is to estimate the parent-specific copy numbers, with 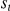 as surrogate information. With the large number of data points obtained from the high density arrays, the posterior for the parent-specific copy numbers is usually quite insensitive to the prior on 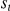. Note that for platforms, such as the Affymetrix 6.0 array, have non-polymorphic copy number markers rather than SNP markers. For those markers, the prior for 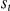 can be set to 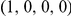. In this way, the posterior will always remain at 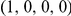 and only the total copy number information at these markers would contribute to the overall segmentation.

Note that this model contains many assumptions, including Gaussianity of the allele specific intensities and Markovicity of the underlying copy number states. These assumptions allow fast and explicit analytic formulas to be derived, thus avoiding the need for Monte Carlo based estimates. For most platforms, the allele-specific intensities deviate from Gaussianity, despite careful normalization. Also, there has never been proof that chromosomal breakages are Markovian. These assumptions are made for modeling convenience, just as in the total-copy number estimation problem [11], [16], [30], [37]. It is reassuring that the estimation method is robust to deviations from both the Gaussian and Markov assumptions, as we show using the titration data from Staaf et al. [35] and through our own spike-in studies.

Our primary objective is to estimate the parent specific copy numbers 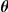, which depend on the observed signals through the unobserved inherited allele configurations 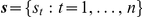. Let 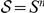 and 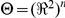 be the set of all possible realizations for 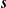 and 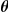, respectively. We describe below an iterative algorithm to estimate 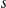 and 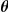.

#### Allele-specific iterative smoothing

Fix stopping threshold 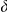. Initialize 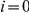 and 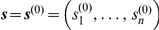 through an initial 4-group clustering of 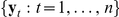. Repeat:

1. Expectation step: Given 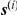, set 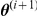 to its posterior mean
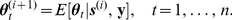
(3)Computationally efficient formulas for (3) are given in Methods.
2. Maximization step: Given 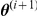, set 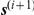 to its maximum a posterior value
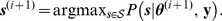
(4)This can be done easily because given 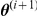, 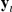 is a four-component mixture of Gaussians at each 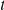, and 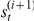 is simply the identifier for each mixture component. The exact formula for (4) is given in Methods.
3. If 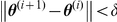, stop and report 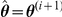, 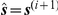. Otherwise, set 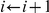 and go back to step 1.

In each iteration of the above algorithm, the expectation step estimates 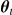 by its posterior mean given the data and the current estimate of the configuration states 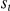. Then, 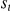 is set to its posterior mode given the data and the current estimate of 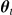. Computationally efficient forward-backward equations for (3) and formulas for (4) are given in Methods, where we also describe an expectation maximization procedure for estimating the hyperparameters 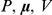, and 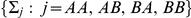 from the data, so that they do not need to be specified a priori.

The above algorithm returns a soft segmentation of 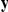 in the form of a Bayesian estimate 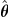 for the parent specific copy numbers at each location. A hard segmentation is sometimes desirable, for example, to give a sparse representation of the data. A hard segmentation can be obtained from the soft segmentation as follows: Compute for each 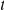 the one-step Euclidean distance 
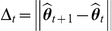
. Estimate the change-points to be the locations where 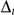 are larger than the threshold, with the constraint that they must be separated by a pre-chosen minimum number of SNPs (e.g. 20). The segmentation algorithm starts with the set 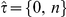 containing only the end points of the sequence. Change-points are added recursively to the set by maximizing 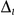 under the separation constraint, until no more change-point can be added. We start with a low threshold for 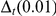 allowing some false positives, with most of the false positives eliminated by a subsequent Wilcoxon Rank-Sum test (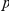-value threshold of 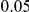) that combines adjacent segments with no significant difference in mean. We found this to be more accurate than a one-step procedure using a more stringent threshold on 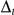.

### Identifying the Type of Aberration

The segmentation divides the genome into regions where the copy numbers of the two inherited chromosomes are constant. It is often useful to know, for each region, whether the copy numbers of one or both parental chromosomes deviate from the normal level. This involves classifying each region into one of the following six types of chromosomal change: gain/gain, gain/normal, balanced gain/loss, unbalanced gain/loss, normal/loss and loss/loss.

For each segmented region, we define the major copy number to be the normalized raw copy number of the more abundant chromosome, and the minor copy number to be the normalized raw copy number of the less abundant chromosome. If the two chromosomes have equal copy numbers, then the major and minor chromosome labels are assigned arbitrarily. The major and minor copy numbers are estimated after the hard-segmentation using a mixture model on the heterozygous SNPs in each region (which can be identified using 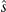). Then, a 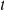-test is used to compare the estimated major and minor copy numbers of each region to the estimated allele copy number of the normal level in the unchanged segments. The Bonferroni correction is used to adjust for multiple testing. The technical details are given in Methods. This procedure allows us to discover and distinguish all of the six types of CNVs.

An additional caveat is that when both parental chromosomes carry the same haplotype, a balanced gain/loss would be called if the region were long enough. Without matched data from normal tissue, it is impossible to distinguish with certainty between inherited and somatic LOH. However, we rely on the fact that long regions of LOH are infrequent, and thus the minor allele frequency of SNPs and the linkage disequilibrium between them can be used to conduct a test for the probability that an inherited LOH appears by chance. This haplotype correction only takes care of the unique common haplotypes, i.e., when a region is dominated by one haplotype. If a haplotype is not common in that region, or if there are several haplotypes in that region, this test loses sensitivity. In this case, paired normal cell information would be useful. More details are given in Methods.

### Results on Simulated Dilution Data from Staaf et al. [35]

Staaf et al. [35] performed a systematic comparison of existing methods for allele-specific copy number estimation. They created a simulated dilution data set based on experimental 550k Illumina data for HapMap sample NA06991. To the diploid HapMap sample, ten regions of aberrant copy number were added at increasing fractions to mimic a tumor sample that is contaminated with normal cells. Here, 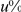 normal cell contamination means 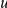 part normal cells are mixed with 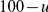 part tumor cells. The aberrant regions vary by type and length, and represent regions of hemizygous gains and losses and copy neutral LOH. Since the locations of the true aberrant regions are known, the specificity and sensitivity of the detection methods can be evaluated.

**Figure 3:**
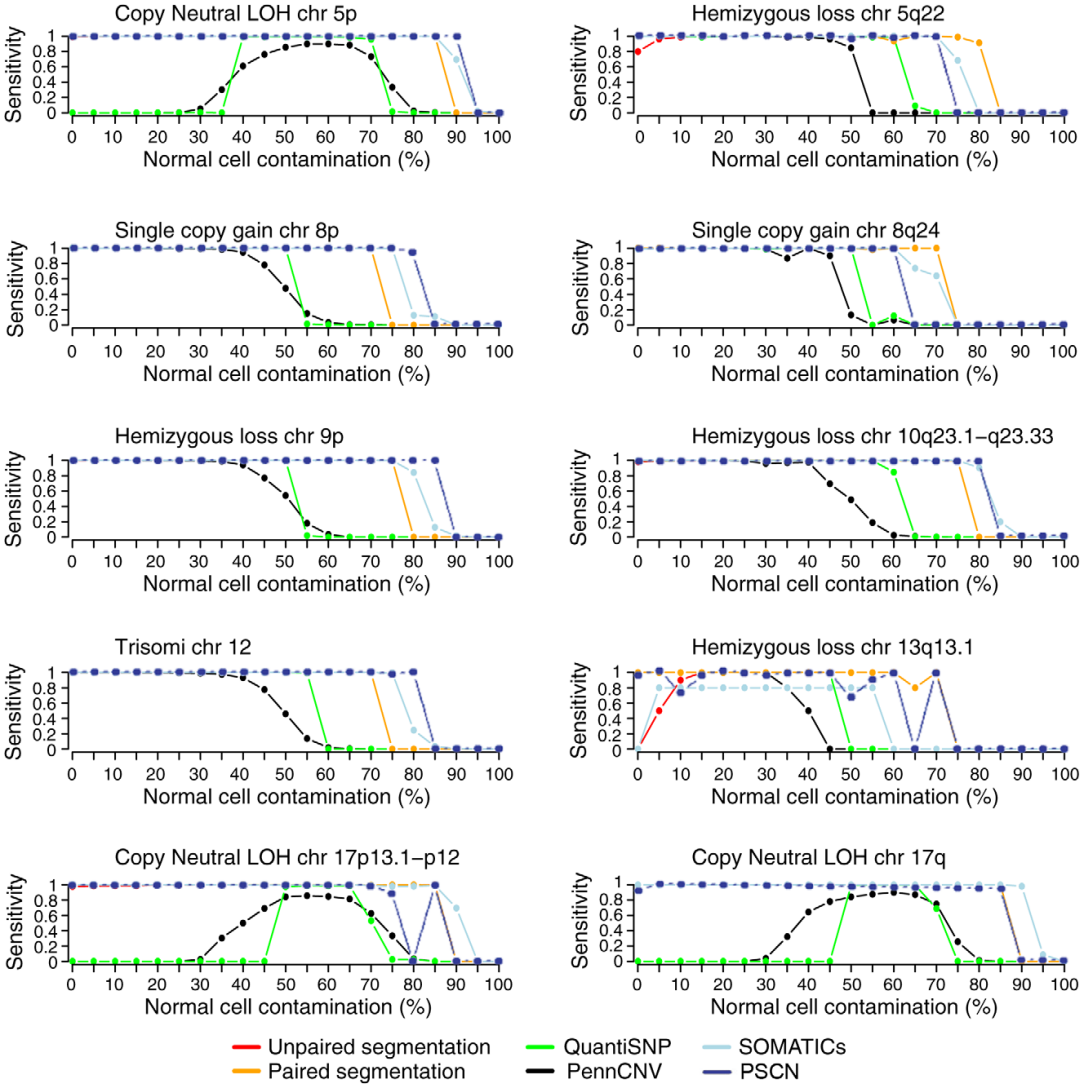
Sensitivity versus normal cell contamination for 10 regions in the dilution data set of Staaf et al. [35] . We overlayed our results on top of plots reproduced from [35].

**Figure 4:**
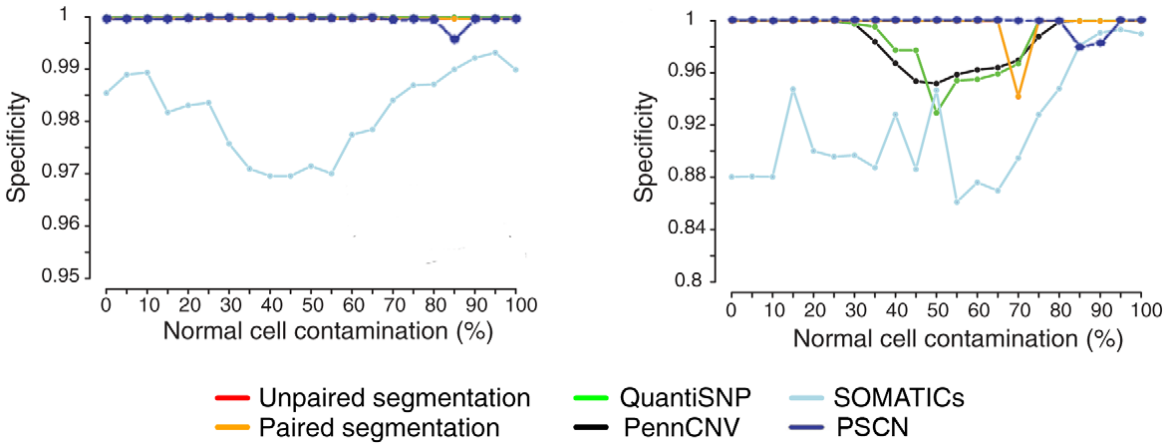
Specificity versus normal cell contamination in the dilution data set of Staaf et al. [35] . We overlayed our results on top of plots reproduced from [35]. Left panel shows the overall specificity, which is the fraction of SNPs outside of all simulated alletic imbalances that are not called. The right panel shows the specificity of correct calling of the type of allelic imbalance, i.e., if a truly aberrant region is identified as aberrant, but of an incorrect type, then it is also considered as a wrong call. Note that the scale of y-axis is different for the two plots.

We applied PSCN, the R package we developed based on our method, to this dilution data set and compared it with existing approaches in an analysis that parallels the insightful analysis in Staaf et al. [35]. The sensitivity and specificity of results from PSCN at varying contamination ratios is shown in Figures 3 and 4 overlayed onto plots reproduced from Staaf et al. [35]. In order to compare with the sensitivity analysis of other models done in the paper by Staaf et al. [35], we define a “correct detection” to mean that a true CNA region has been called, but do not require that the type of CNA (e.g. gain/loss, normal/loss) has been correctly identified. All the other current procedures only categorize the CNAs into Gain, Loss and LOH, which are the three types of CNAs used in the Dilution data in Staaf et al. [35]. We assess the accuracy of PSCN in a more detailed classification of identified CNAs based on the six types of chromosomal change in a separate data set that contains a wider diversity of chromosomal events (see next section). In the simulated dilution data, the regions vary in length, magnitude, and type of aberration, with some regions harder to detect than the others. There is a separate sensitivity plot for each of the 10 aberrant regions created by [35]. As expected, for all regions, sensitivity is maintained at a high level up to a certain contamination ratio, then drops sharply. Since Staaf et al. and we used very stringent detection thresholds, the specificity is maintained near 1 for all contamination ratios, as shown in Figure 4. The sensitivity of PSCN is comparable to SOMATICs [36], but the latter method has much lower specificity, as shown in the analysis of Staaf et al., see Figure 4. PSCN achieves good accuracy compared to the other existing methods, especially methods based on discrete-state hidden Markov models for high levels of contamination. The discrepancy between the two specificity plots in Figure 4 are due to the fact that when an aberration is called, it may be labeled as an incorrect type (for example, a copy neutral LOH may be labeled as single copy gain). When the correct calling of aberration type is required, the specificity of PSCN is maintained through a higher level of contamination as compared to existing models. The new model can identify the correct aberration type if the normal cell contamination is below 80%. Above 80%, PSCN gains significantly in sensitivity compared to existing methods but also sacrifices slightly in specificity.

### Accuracy of Aberration Type Identification

**Figure 5:**
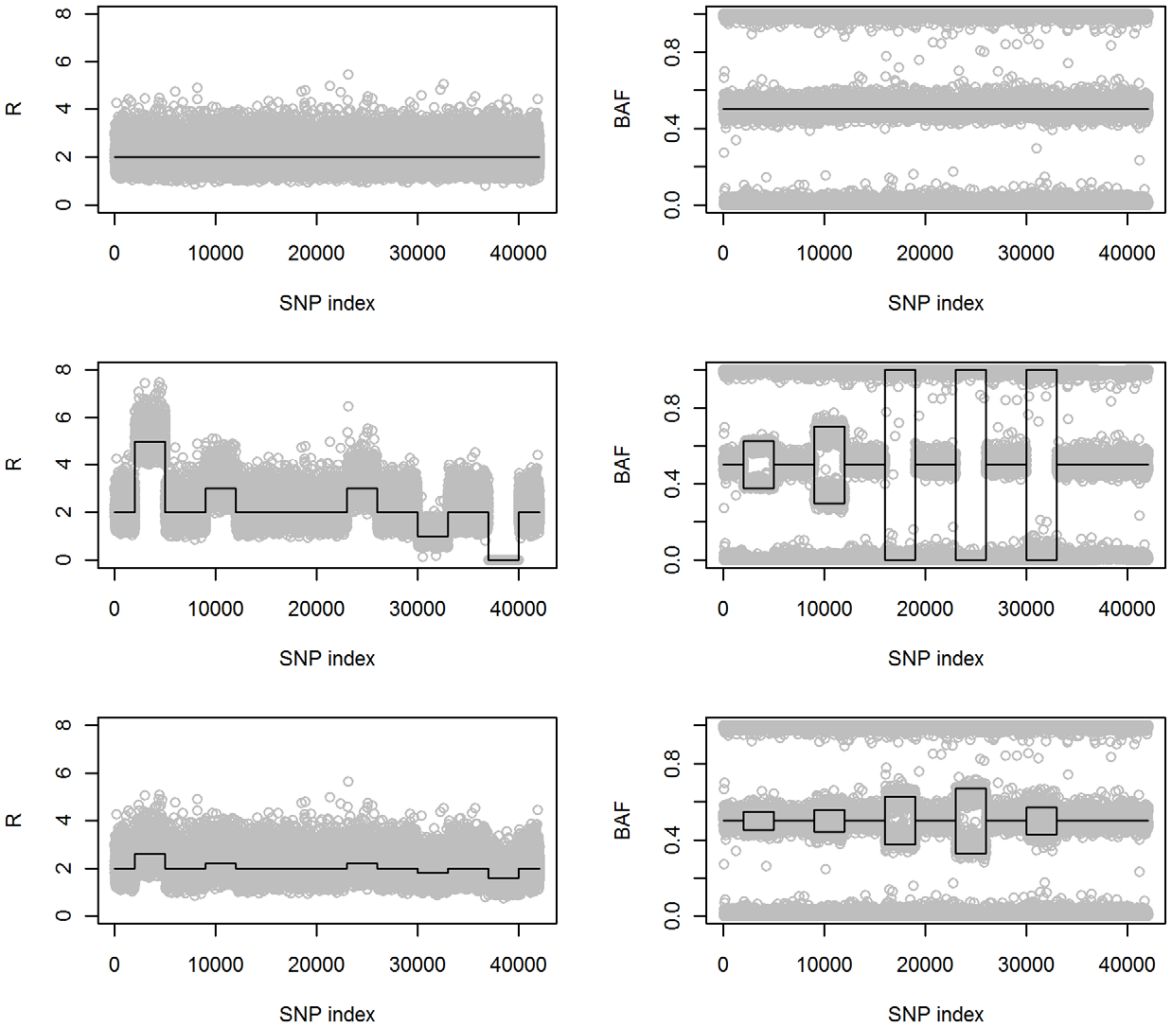
Signal of the simulated data by imposing six types of aberrations on chromosome 1 of HapMap sample NA06991. The first row shows 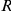 and BAF before the signals are imposed. The second row shows 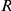 and BAF after the signals are imposed, under normal cell contamination 0%. True signals are indicated by black lines. The third row shows 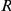 and BAF after the signals are imposed, under 80% normal cell contamination.

**Figure 6:**
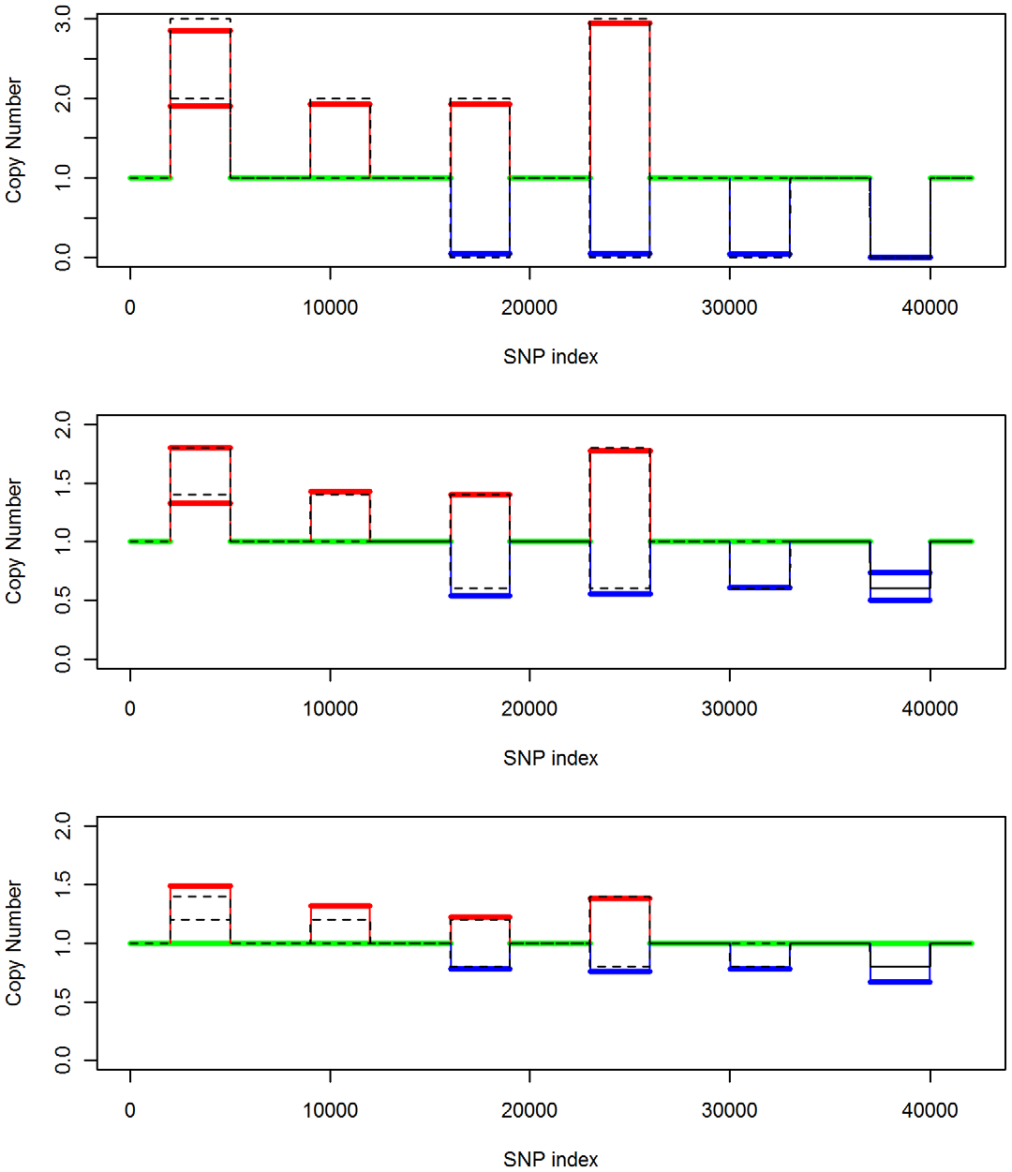
Copy number estimation of PSCN on the simulated data by imposing six types of aberrations on chromosome 1 of HapMap sample NA06991. Top panel: no normal cell contamination. Middle panel: normal cell contamination 60%. Bottom panel: normal cell contamination 80%. In all panels, solid lines denote estimated allele copy numbers and dashed lines denote true copy numbers.

The dilution data set from Staaf et al. [35] contains only three types of aberrations: hemizygous loss (normal/loss), single copy gain (gain/normal), and copy neutral LOH (balanced gain/loss). We created a simulated data set containing all six types of aberrations: gain/gain, gain/normal, balanced gain/loss, unbalanced gain/loss, normal/loss and loss/loss. To make the simulation resemble real data, we started with the 550k Illumina data for chromosome 1 of HapMap sample NA06991. To this normal sequence we imposed six different signal types on six regions. The positions and magnitudes of the added signals are shown in Table 2. The top panel of Figure 5 (first row) shows the 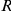 and BAF before the signals are imposed. The middle and bottom panels show the 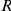 and BAF after the signals have been imposed, at 0% and 80% contamination respectively, with true signals indicated by black lines. Signal becomes weaker when normal cell contamination increases, and thus are harder to detect. The estimated parent-specific copy numbers are shown in Figure 6. We can see from the plots that the estimated parent-specific copy numbers are very close to the true allele copy numbers. Table 3 shows the largest normal cell contamination under which the signals are detectable by PSCN. When normal cell contamination is less than 80%, our model can detect most of the signals with both alleles assigned to the correct type. When the normal cell contamination rises to 90%, our model can still detect three out of the six CNA regions, but assigns the correct type to only one of the two alleles. For example, at a high contamination level of 90%, there is a tendency for a fractional loss of both chromosomes to be mistaken for a fractional loss of only one of the two chromosomes. From this study, we see that the correct type of aberration can be identified robustly for all but the highest levels of normal cell contamination.

**Table 2:** Signals imposed on to Chromosome 1.

	SNP begin	SNP end	Major copy number	Minor copy number
Gain/Gain	2000	5000	3	2
Gain/Normal	9000	12000	2	1
Balanced Gain/Loss	16000	19000	2	0
Unbalanced Gain/Loss	23000	26000	3	0
Normal/Loss	30000	33000	1	0
Loss/Loss	37000	40000	0	0

“SNP begin” and “SNP end” are the indices of the SNP where the added signal begins and ends, respectively. “Major” and “minor” copy numbers are the intensities of the signal in the two alleles.

**Table 3:** The largest tolerable percentage for normal cell contamination under which the type of aberration can be correctly detected (left column), and under which the type of aberration can be correctly identified for one of the two alleles when both alleles are different from normal (eg. Gain/Gain identified as Gain/Normal) (right column).

	Correct Type Estimated for both alleles	Correct Type Estimated for one allele
Gain/Gain	70	90
Gain/Normal	85	Not applicable.
Balanced Gain/Loss	80	90
Unbalanced Gain/Loss	85	90
Normal/Loss	85	Not applicable.
Loss/Loss	65	80

All numbers are in percent.

### Accuracy of Estimation of Genotype States

Using the dilution data set created from HapMap sample NA06991, we can also assess the accuracy of PSCN in identifying the genotype states 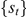. Since the genotypes for the SNPs on this sample are known, we simply compared the estimated 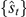 with the true values.

Table 4 shows the percent of homozygous SNPs that are misclassified as heterozygous, and vice versa. When the SNP is classified as homozygous, the determination between the states AA and BB is trivial, and no errors are made. When normal cell contamination is extremely low, less than 10%, genotyping errors are common in regions of loss of heterozygosity (either normal/loss or gain/loss). This is expected, since in a region with complete LOH and zero contamination, only one of the two parental alleles is left, and thus it would be impossible to distinguish between the homozygous configurations 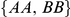 and the heteryzogous configurations 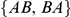. Fortunately, these types of genotyping errors would not affect the accurate estimation of 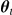, since the mean levels for the heterozygous and homozygous tracks merge for LOH regions under zero contamination. It is slightly unintuitive that the correct estimation of 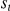 depends on the fact that there is normal cell contamination! This is reflected in Table 4, where accuracy quickly improves as normal cell contamination increases, with a total misclassification rate of 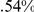 at 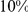 normal cell contamination.

**Table 4:** The number of misclassifications of each type in the identification of 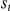 on the NA06991 dilution data set, at different levels of normal cell contamination.

Normal Contamination (%)	Homozygous 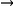 Heterozygous	Heterozygous 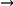 Homozygous	Misclassification Rate (%)
0	1285	2791	9.7
5	986	1	2.3
10	228	0	.54
25	20	0	.048
50	93	0	.22
90	39	0	.093

There are 42037 SNPs total.

A complete analysis of the misclassification rates of 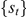 are given in the Supporting Information file (Text S1).

### Analysis of TCGA Glioblastoma Samples

We applied PSCN to 223 glioblastoma samples from the TCGA project [38]. These samples were assayed using Illumina HumanHap 550k SNP arrays.

Almost all of the 223 samples analyzed contain substantial copy number aberrations. Table 5 shows the distribution of the types of copy number events found in the samples. Of the gain/loss events, which comprise 45.4% of all of the events, 22.8% are copy neutral LOH and 22.5% are unbalanced gain/loss. We see from this table that, among these glioblastoma samples, single chromosome losses or single chromosome gains comprise 49.6% of all the events, which means that more than half of the events involve change of both inherited chromosomes.

**Table 5:** Distribution of types of copy number aberrations across all events found in the 223 glioblastoma samples.

Event type	%	count
gain/gain	3.6	1315
gain/normal	21.0	7773
balanced gain/loss	22.9	8568
unbalanced gain/loss	22.5	8352
normal/loss	28.6	10598
loss/loss	1.4	521

**Figure 7:**
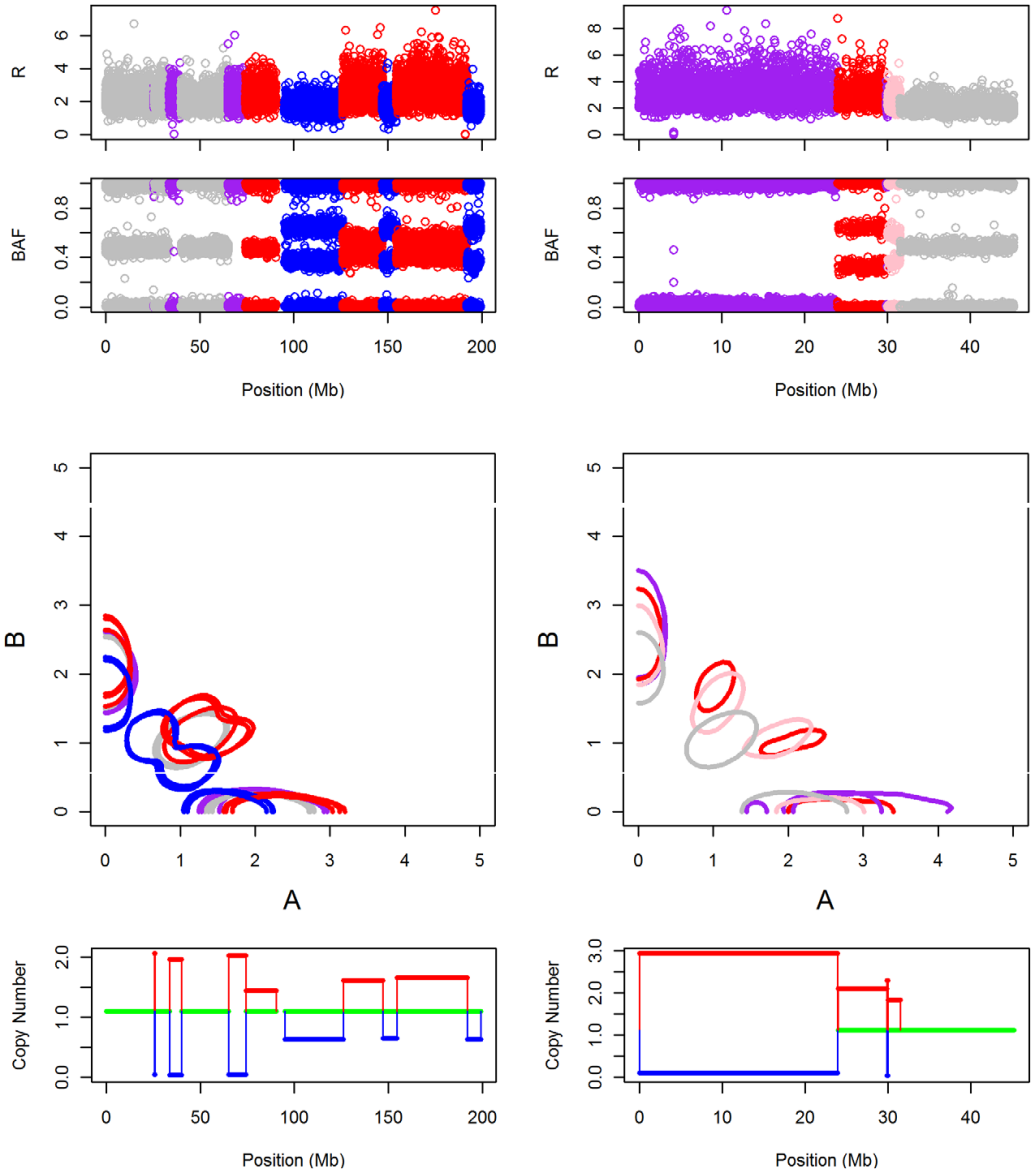
Example regions from TCGA sample 02-0332 chromosome 3 (left) and TCGA sample 02-0258 chromosome 2 (first 10000 SNPs) (right). The plots, in order from the top, show the 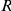 values, BAF values, 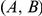 contours and estimated major and minor copy numbers. The top three plots are color coded by the segmentation estimated using our procedure. In the color coding of the bottom plot, red represents gain, blue represents loss, and green represents normal.

We now zoom in on two example regions to illustrate the additional insights gained from parent-specific copy number analysis. These regions are shown in Figure 7. The figures in the left panel correspond to the entire chromosome 3 of TCGA glioblastoma sample 02-0332, while those on the right panel correspond to the first 10000 SNPs on chromosome 2 of TCGA glioblastoma sample 02-0258. The top two plots in each panel show the 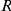 and BAF values. The color scheme for these plots show the segmentation obtained using PSCN. We transformed the 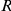 and BAF values back to the 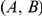 raw copy number values, and fitted two dimensional densities separately to each region in the segmentation. The contours of the two dimensional density estimates, delineating the locations of the clusters, are shown in the third plot from the top in each panel. The color scheme for the contours is the same as the color scheme for the 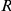 and BAF plots. Finally, the bottom plot of each panel shows the estimated major and minor copy numbers for each region (we will call this type of plot the *mm*-plot). The color scheme of the *mm*-plot reflects the gain/loss status of each region, where red represents gain, blue represents loss, and green represents normal. It is usually difficult to discern the relative magnitudes of gains and losses from the 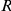 and BAF plots, especially when both inherited chromosomes have undergone copy number changes. Such relative changes in parent specific copy numbers can be quantified more easily by examining the 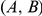 contour and 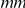-plots.

#### Copy neutral LOH (Balanced Gain/Loss)

First, consider the example region from TCGA sample 02-0332 on the left panel. There are three instances of copy neutral LOH, colored in purple. Based on the BAF plot, the loss seems to be complete, that is, it is carried by almost all of the cells in the sample. The 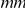-plot also gives this information, as the estimated major copy number (red line) is close to 2, and the estimated minor copy number (blue line) is close to 0. These LOH regions do not change the total copy number, and thus would not have been detected if the segmentation were based on the 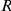 profile. On the other hand, an analysis based only on the BAF plot would not have revealed that the LOH is copy neutral; e.g. in the TCGA sample 02-0258, the LOH region (purple) with similar pattern in BAF is not copy neutral. The estimates in the 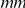-plot can only be obtained through a joint analysis of both the 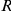 and the BAF profiles.

#### Fractional single chromosome gains and losses

Following the copy neutral LOH regions in chromosome 3 of sample 02-0332, there is a stretch of alternating gains and losses, colored respectively in red and blue. The copy of the other parental chromosome in these regions is one. As seen from the 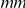-plot, all of these regions contain changes that affect only one of the two inherited chromosomes. The changed chromosome may differ across segments. For example, the paternal chromosome may have been differed in one segment, and the maternal chromosome in the next. The copy number of the other chromosome in these regions remain at the normal level. This fact can not be deduced from total copy number analysis, as an increase in 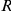 can be due to gains of both inherited chromosomes, or an unbalanced gain of one chromosome and loss of the other; see the next example (TCGA 02-0258). The 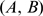 contour plot discriminates between these two possible cases. If we examine the cluster centers corresponding to the heterozygotes in the red and blue segments we see that for any one cluster, only one of the 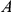 and 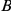 coordinates is significantly shifted from the corresponding coordinate of the normal AB cluster (coded in gray). This is evidence that the copy number of only one of the chromosomes has changed in these regions. The positions of the heterozygote cluster centers of the red and blue regions indicate only a partial gain and loss, as their shifts from normal are only a fraction of that expected in a complete event. The estimated major and minor copy numbers in the 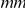-plot quantifies the partial change explicitly, with the major copy numbers at around 1.5 for the gain and the minor copy numbers at around 0.5 for the loss. Assuming a linear signal response curve for the Illumina platform in the range between 0 and 3 fold change in DNA quantity, this translates to about 50% of the cells in the tumor sample carrying the aberrations coded in blue and red.

The same reasoning can be applied to the red and pink regions of chromosome 2 of TCGA sample 02-0258 (right panel), which contains a fractional gain. By teasing apart the copy numbers of each inherited chromosome, we are now able to characterize and quantify these fractional changes.

#### Simultaneous unbalanced gain and loss of both chromosomes (unbalanced gain/loss)

Now consider the example region color coded in purple from TCGA sample 02-0258 in the right panel. The 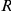 plot suggests that there is a gain in total copy number. However, the BAF plot reveals that there seems also to be an almost complete loss of heterozygosity in this region. Loss of one of the inherited chromosomes is necessary for loss of heterozygosity. Thus we conclude that the region colored in purple contains both a gain of one as well as an almost complete loss of the other inherited chromosome. Indeed, as the 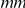-plot shows, the estimated major and minor copy number fold changes for this region have values of 3 and 0, respectively. The gain and loss of the two inherited chromosomes is thus unbalanced, suggesting that this region may have experienced multiple mutations. This region is immediately followed by a gain of only one of the two inherited chromosomes (see the 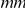-plot), of magnitude roughly equal to the difference between the deviations of the major and minor copy numbers from normal. This suggests the hypothesis that this sample first experienced a gain of one of the inherited chromosomes that covered the purple and red regions, then a LOH which caused a gain of the already amplified chromosome and a simultaneous loss of the other inherited chromosome. Our analysis of the TCGA data shows that these types of unbalanced gain and loss events are quite common.

## Discussion

We have developed a method for simultaneous estimation of parent-specific DNA copy number and inherited genotypes for tumor samples using allele-specific raw copy number data. The model and estimation procedure start with transforming allele-specific data into 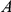 and 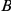 intensities, which may vary across experimental platforms. The model assumes that the 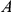 and 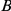 allele intensities should be roughly symmetric, roughly variance stabilized and have approximately bivariate Gaussian errors. Indeed, the model is quite robust to the violation of the bivariate Gaussian error assumption. The model gives satisfying results even if this assumption is heavily violated. More details are shown in the the Supporting Information file (Text S1). We illustrated the method and evaluated its performance on both published and newly generated dilution data sets on the Illumina platform.

A rigorous assessment using in silico titration data provided by Staaf et al. [35] shows that PSCN has good accuracy. The proposed method does not require paired normal samples. However, if such samples were available, then they can be used to further improve accuracy and to distinguish between inherited LOH and somatic LOH. In such cases, 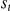 can simply be set to the genotypes inferred from the normal samples.

PSCN is not platform specific, and we have also applied it to data from the Affymetrix Genotyping 6.0 array, with an example analysis given in the Supporting Information file (Text S1). The segmentation accuracy of PSCN seems to be reasonable for Affymetrix data, but can potentially be improved significantly by better probe-level normalization. This is due to the fact that the BAF of Affymetrix data is much noisier than the BAF of Illumina data, which makes the estimation of 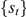 much more difficult. Bengtsson et al. [39] have shown that much of the variation in the BAF of Affymetrix data are due to probe-specific effects that can be removed if a matched normal sample is available. Another promising method for probe-level normalization of Affymetrix data is the probe raw copy number composite representation (PICR) model of Wan et al. [29], which uses probe sequence information and physico-chemical modeling to estimate binding affinity. However, since the PICR model relies on mismatch probes, it is only applicable to Affymetrix platforms prior to the 6.0 array. Thus, better probe-level normalization of Affymetrix 6.0 data for unmatched samples is still an important problem for further investigation.

An overview of an analysis of the TCGA glioblastoma samples reveal that a substantial fraction of copy number changes are copy-neutral loss of heterozygosity events. These events would not have been found using analyses based only on total copy number. Cases of unbalanced simultaneous changes in the copy numbers of both inherited chromosomes were also found. It would be of interest to quantify the frequency of such changes among different cancer subtypes and in other types of tumors.

A final point that we would like to emphasize is the quantification of fractional changes, as exemplified by the two case studies on the TCGA glioblastoma samples. Since this requires teasing apart the quantities of the two inherited chromosomes, it can only be achieved through allele-specific estimates. The fraction of cells that carry each copy number event is important for downstream analyses, such as quantifying normal cell contamination and studying tumor microevolution. The parent-specific copy number estimates obtained from the proposed method provides a starting point for these types of investigations.

The R package for PSCN is registered on R-Forge (http://r-forge.r-project.org/) under project name PSCN.

## Methods

### Data Transformation

The proposed model is not platform specific, and can theoretically be applied to any type of allele-specific copy number data where the errors on the raw copy number values of the alleles can be normalized to approximately adhere to a bi-variate Gaussian distribution. As we show below, the Gaussian error assumption allows for explicit analytic formulas for the posterior mean of the underlying inherited chromosome copy numbers, thus bypassing the need for computationally intensive Monte Carlo methods. For most platforms, the raw allele-specific raw copy number values must be properly normalized for this error model to be a good approximation. However, as we mentioned in the Discussion section, the model is quite robust to the violation of the Gaussian error assumption.

A unified approach that gives satisfying results for data from both Illumina and Affymetrix platforms is as follows. Since
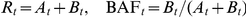
we have
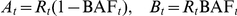
Note that the “BAF” given by the Illumina platform [6] is not the intuitive quantity (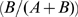), but the arc-tangent of the ratio of 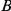 raw copy number versus 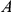 raw copy number scaled to [0,1]. Use 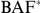 to denote the so called BAF given by Illumina, then
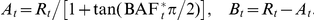
For PSCN we use 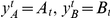.

### Explicit formulas for 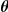 given 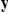 and 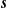


We give here exact formulas for the conditional expectation (3). Let 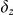 denote the probability distribution that assigns probability 1 to the value 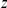. Denote by 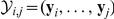, and 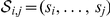. A brief outline of the estimation procedure is as follows: First, conditioned on all data to the left of 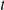, 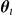 is distributed as a mixture of Gaussians:
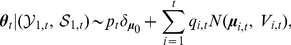
(5)where the formulas for computing the parameters of the mixture 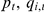, 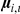, and 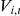 are given below. We call (5) the forward filter. Since by our model 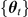 is a reversible Markov chain, we can reverse time and obtain a backward filter that is analogous to (5):
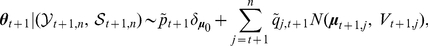
(6)where the parameters 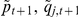, 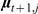, and 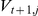, as for the forward filter, are given in explicitly computable form below. The Bayes theorem can then be used to combine the forward filter (5) and backward filter (6) to derive the posterior distribution of 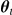 given the complete sequence 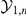, which is a mixture of normal distributions
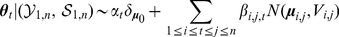
(7)whose parameters can be derived from the forward and backward filters as described below. This forward-backward procedure can be reduced to 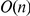 computation time by the BCMIX algorithm [40]. From (7), it follows that the conditional expectation in Equation (3) can be computed as
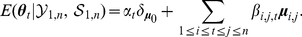
(8)


#### The forward filter

Let 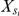 be allele assignment matrices depending on 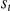:
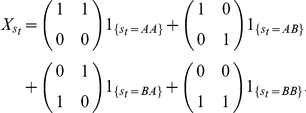
Let 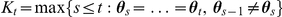 denote the nearest change-point at a location less than or equal to 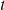. Define
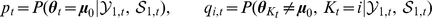
for 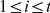. The conditional distribution of 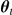, given 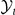 and the event that 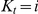 and 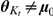, is 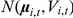, where
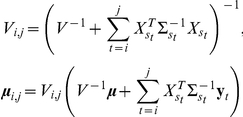
for 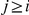. It follows that the posterior distribution of 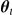 given 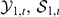 is the mixture of normal distributions and a point mass at 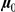 given by (5). Let 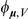 denote the density function of the 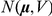 distribution, i.e.,
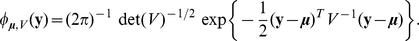
Making use of 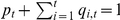, it is possible to show as in Lai et al. [37] that the conditional probabilities 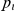 and 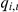 can be determined by the recursions
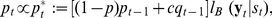
(9)

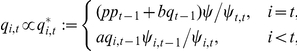
where 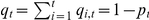, 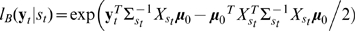, 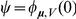 and 
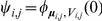
 for 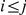. Specifically, the mixture probabilities in (5) are 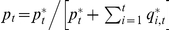 and 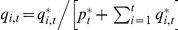.

#### The smoothing estimate

Since 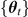 is a reversible Markov chain, we can reverse time and apply the same steps as in the forward equations to obtain (6), in which the weights 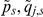 can be obtained by backward induction using the time-reversed counterpart of (9):
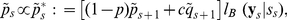
(10)

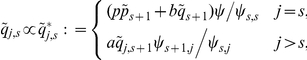
where 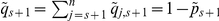. Since for any set 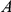, 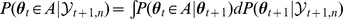, it follows from (6) and the reversibility of 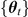 that
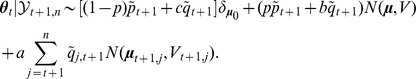
The recursions for deriving the components of the mixture for (7) are exactly the same as those for the earlier model limited to total copy number in Lai et al. [37]:
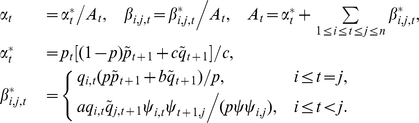
and we refer the reader to Lai et al. [37] for their derivation.

### Estimation of 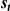


The variables 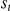 are assumed to be i.i.d., with
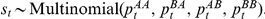
The inherited allele configurations 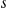 is assumed to be independent of 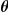, so
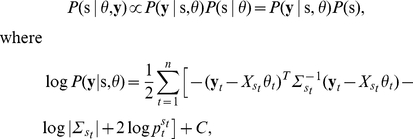
(11)where 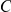 is a constant. Each component of the above sum can be maximized separately to give, for each 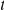,
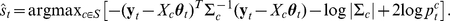



### Region Characterization

Let 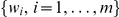 be 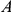 and 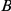 intensities of heterozygous SNPs for segments at normal state and 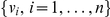 be 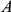 and 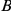 intensities of heterozygous SNPs for the segment being tested. Then, 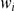, 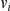 follow the model:
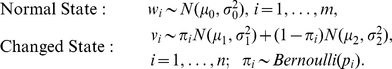
For the normal state, we can estimated the parameters easily as
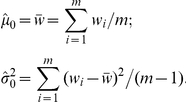
For the target segment, 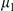, 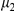, 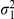, 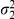 can be estimated by EM algorithm:

**Step 1:** Initialize: 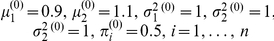


**Step 2:** Set
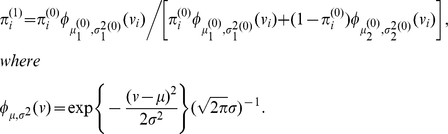



**Step 3:** Set
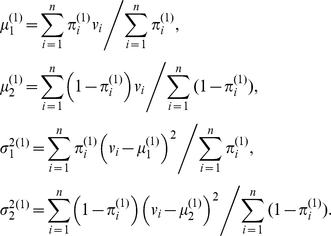



**Step 4:** Stop if 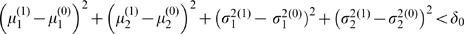, where 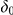 is a pre-chosen threshold (PSCN has default value 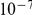). Otherwise, set 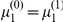, 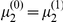, 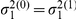, 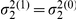, and go back to step 2.

The motivation of the initial and default settings are as follows. For segment with changed states, the goal is to estimate minor and major copy number. It is expected that the minor copy number would be less than or equal to 1 and the major copy number would be larger than or equal to 1, so the initial values for 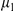 and 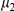 are set to 0.9 and 1.1 respectively. Although it is possible that both chromosomes in a segment are gained or lost, a small discrepancy of the initial values of 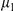 and 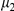 will also be a good start. Also, it is expected that the numbers of AB and BA states in a segment is similar, so the initial value of 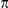 is set to 0.5. The initial values for 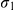 and 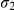 can be quite arbitrary, with 1 being a reasonable value to use. 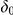 is set to be 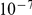, which is small enough to indicate a convergence of the iterative algorithm.

Denote the estimated parameters by 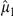, 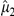, 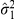, 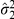, 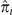. To test the hypothesis 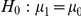, the standard 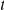-statistic is
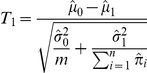
Under 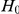, the distribution of 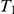 is 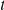 with degree of freedom 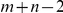, so 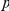-value can be calculated and compared with the level of the test. The null hypothesis that 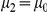 needs also be tested, by replacing 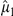 with 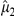 in the above equation.

## Supporting Information

Text S1 Supporting materials for PSCN.
(0.28 MB PDF)
