# Supplementary material for: Estimation of Parent Specific DNA Copy Number in Tumors using High-Density Genotyping Arrays
Source: PLoS Comput Biol. 2011 Jan 27;7(1):e1001060. doi: 10.1371/journal.pcbi.1001060 (PMC3029233; doi:10.1371/journal.pcbi.1001060)
Supplement: Text S1 — Supporting materials for PSCN. (0.28 MB PDF) [file pcbi.1001060.s001.pdf]

Supporting Information file (Text S1)

Hao Chen, Haipeng Xing, and Nancy R. Zhang

1 EM Algorithm for Hyperparameter Estimation

We use the same notation as in the main manuscript. For simplicity, $\mathcal{Y}_t$ and $\mathcal{S}_t$ are used as shorthand for $\mathcal{Y}_{1,t}$ and $\mathcal{S}_{1,t}$, respectively. Extending the arguments in Lai et al. [1], we can show that the conditional density function of $\mathbf{y}_t$ given $(\mathcal{Y}_{t-1}, \mathcal{S}_n)$ is

$$f(\mathbf{y}_t|\mathcal{Y}_{t-1}, \mathcal{S}_n) = \big(p_t^* + \sum_{i=1}^{t} q_{i,t}^*\big)\phi_{0,\sigma^2}(\mathbf{y}_t), \tag{1}$$

where p_t^* and $q_{i,t}^*$ are given by (11) and are functions of the hyperparameter vector $\Phi = (p, b, c, \boldsymbol{\mu}, V, \Sigma_{AA}, \Sigma_{AB}, \Sigma_{BA}, \Sigma_{BB})$. Given Φ and the observed data $(\mathcal{Y}_n, \mathcal{S}_n)$, the log likelihood function is

$$l(\Phi|\mathcal{S}_n) = \sum_{t=1}^{n} \log f(\mathbf{y}_t|\mathcal{Y}_{t-1}, \mathcal{S}_n) = \sum_{t=1}^{n} \log\Big\{\big(p_t^* + \sum_{i=1}^{t} q_{i,t}^*\big)\phi_{0,\sigma^2}(\mathbf{y}_t)\Big\}, \tag{2}$$

in which $f(\cdot|\cdot)$ denotes conditional density function. Maximizing (2) over Φ yields the maximum likelihood estimate $\widehat{\Phi}$.

Since Φ is a 20-dimensional vector and the functions $p_t^*(\Phi)$ and $q_{i,t}^*(\Phi)$ have to be computed recursively for $1 \le t \le n$, direct maximization of (2) may be computationally expensive due to the curse of dimensionality. An alternative approach is to use the EM algorithm which exploits the much simpler structure of the log likelihood $l(\Phi|\mathcal{S}_n)$ of the data $\{(\mathbf{y}_t, \boldsymbol{\theta}_t), 1 \le t \le n\}$:

$$\begin{aligned} l(\Phi|\mathcal{S}_n) \;=\; & -\frac{1}{2}\sum_{t=1}^{n}\Big\{(\mathbf{y}_t - X_{s_t}\boldsymbol{\theta}_t)'\Sigma_{s_t}^{-1}(\mathbf{y}_t - X_{s_t}\boldsymbol{\theta}_t) + \log|\Sigma_{s_t}| + 2\log(2\pi)\Big\} \\ & -\frac{1}{2}\sum_{t=1}^{n}\Big\{(\boldsymbol{\theta}_t - \boldsymbol{\mu})'V^{-1}(\boldsymbol{\theta}_t - \boldsymbol{\mu}) + \log|V| + 2\log(2\pi)\Big\}\mathbf{1}_{\{\boldsymbol{\mu}_0 \neq \boldsymbol{\theta}_t \neq \boldsymbol{\theta}_{t-1}\}} \\ & + \sum_{t=1}^{n}\Big\{[\log(1-p)]\mathbf{1}_{\{\boldsymbol{\theta}_t = \boldsymbol{\theta}_{t-1} = \boldsymbol{\mu}_0\}} + (\log p)\mathbf{1}_{\{\boldsymbol{\theta}_t \neq \boldsymbol{\theta}_{t-1} = \boldsymbol{\mu}_0\}}\Big\} \\ & + \sum_{t=1}^{n}\Big\{[\log(1-b-c)]\mathbf{1}_{\{\boldsymbol{\theta}_t = \boldsymbol{\theta}_{t-1} \neq \boldsymbol{\mu}_0\}} + (\log c)\mathbf{1}_{\{\boldsymbol{\theta}_t = \boldsymbol{\mu}_0 \neq \boldsymbol{\theta}_{t-1}\}} + (\log b)\mathbf{1}_{\{\boldsymbol{\mu}_0 \neq \boldsymbol{\theta}_t \neq \boldsymbol{\theta}_{t-1} \neq \boldsymbol{\mu}_0\}}\Big\}. \end{aligned} \tag{3}$$

Since $l(\Phi|\mathcal{S}_n)$ decomposes into normal and multinomial components, the E-step of the EM algorithm involves $E\big((\boldsymbol{\theta}_t - \boldsymbol{\mu})(\boldsymbol{\theta}_t - \boldsymbol{\mu})'|\mathcal{Y}_n, \mathcal{S}_n\big)$, $E\big((\mathbf{y}_t - X_{s_t}\boldsymbol{\theta}_t)(\mathbf{y}_t - X_{s_t}\boldsymbol{\theta}_t)'|\mathcal{Y}_n, \mathcal{S}_n\big)$ and the conditional probabilities

$$P(\boldsymbol{\theta}_t = \boldsymbol{\mu}_0 = \boldsymbol{\theta}_{t-1}|\mathcal{Y}_n, \mathcal{S}_n) = \frac{(1-p)p_{t-1}\alpha_t}{(1-p)p_{t-1} + cq_{t-1}},$$

$$P(\boldsymbol{\theta}_t = \boldsymbol{\mu}_0 \neq \boldsymbol{\theta}_{t-1}|\mathcal{Y}_n, \mathcal{S}_n) = \frac{cq_{t-1}\alpha_t}{(1-p)p_{t-1} + cq_{t-1}},$$

$$P(\boldsymbol{\theta}_t \neq \boldsymbol{\theta}_{t-1} = \boldsymbol{\mu}_0|\mathcal{Y}_n, \mathcal{S}_n) = c\widetilde{q}_t\alpha_{t-1}\Big/\{(1-p)\widetilde{p}_t + c\widetilde{q}_t\},$$

$$P(\boldsymbol{\mu}_0 \neq \boldsymbol{\theta}_t \neq \boldsymbol{\theta}_{t-1} \neq \boldsymbol{\mu}_0|\mathcal{Y}_n, \mathcal{S}_n) = (\sum_{j=t}^{n} \beta_{t,j,t})bq_{t-1}\Big/\{bq_{t-1} + pp_{t-1}\},$$

together with $P(\boldsymbol{\theta}_t = \boldsymbol{\theta}_{t-1} \neq \boldsymbol{\mu}_0|\mathcal{Y}_n, \mathcal{S}_n)$, which is determined by the property that those five conditional probability have to sum up to 1. In view of (3), the M-step of the EM algorithm involves the closed-form updating formulas

$$\begin{aligned}
1-\widehat{p}_{\text{new}} &= \big[\Sigma_1^n P(\boldsymbol{\theta}_t = \boldsymbol{\theta}_{t-1} = \boldsymbol{\mu}_0|\mathcal{Y}_n, \mathcal{S}_n, \widehat{\Phi}_{\text{old}})\big]\Big/\big[\Sigma_1^n P(\boldsymbol{\theta}_{t-1} = \boldsymbol{\mu}_0|\mathcal{Y}_n, \mathcal{S}_n, \widehat{\Phi}_{\text{old}})\big],\\
\widehat{a}_{\text{new}} &= \big[\Sigma_1^n P(\boldsymbol{\theta}_t = \boldsymbol{\theta}_{t-1} \neq \boldsymbol{\mu}_0|\mathcal{Y}_n, \mathcal{S}_n, \widehat{\Phi}_{\text{old}})\big]\Big/\big[\Sigma_1^n P(\boldsymbol{\theta}_{t-1} \neq \boldsymbol{\mu}_0|\mathcal{Y}_n, \mathcal{S}_n, \widehat{\Phi}_{\text{old}})\big],\\
\widehat{c}_{\text{new}} &= \big[\Sigma_1^n P(\boldsymbol{\theta}_t = \boldsymbol{\mu}_0 \neq \boldsymbol{\theta}_{t-1}|\mathcal{Y}_n, \mathcal{S}_n, \widehat{\Phi}_{\text{old}})\big]\Big/\big[\Sigma_1^n P(\boldsymbol{\theta}_{t-1} \neq \boldsymbol{\mu}_0|\mathcal{Y}_n, \mathcal{S}_n, \widehat{\Phi}_{\text{old}})\big],\\
\widehat{\boldsymbol{\mu}}_{\text{new}} &= \big[\Sigma_1^n E(\boldsymbol{\theta}_t \mathbf{1}_{\{\boldsymbol{\mu}_0 \neq \boldsymbol{\theta}_t \neq \boldsymbol{\theta}_{t-1}\}}|\mathcal{Y}_n, \mathcal{S}_n, \widehat{\Phi}_{\text{old}}\big]\Big/\big[\Sigma_1^n P(\boldsymbol{\mu}_0 \neq \boldsymbol{\theta}_t \neq \boldsymbol{\theta}_{t-1}|\mathcal{Y}_n, \mathcal{S}_n, \widehat{\Phi}_{\text{old}})\big],\\
\widehat{V}_{\text{new}} &= \big[\Sigma_1^n E\{(\boldsymbol{\theta}_t - \widehat{\boldsymbol{\mu}}_{\text{old}})(\boldsymbol{\theta}_t - \widehat{\boldsymbol{\mu}}_{\text{old}})'\mathbf{1}_{\{\boldsymbol{\mu}_0 \neq \boldsymbol{\theta}_t \neq \boldsymbol{\theta}_{t-1}\}}|\mathcal{Y}_n, \mathcal{S}_n, \widehat{\Phi}_{\text{old}}\}\big]\\
&\quad \cdot\big[\Sigma_1^n P(\boldsymbol{\mu}_0 \neq \boldsymbol{\theta}_t \neq \boldsymbol{\theta}_{t-1}|\mathcal{Y}_n, \mathcal{S}_n, \widehat{\Phi}_{\text{old}})\big]^{-1},\\
\widehat{\Sigma}_{AA,\text{new}} &= \Sigma_{t=1}^n E\big[(\mathbf{y}_t - X_{s_t}\boldsymbol{\theta}_t)(\mathbf{y}_t - X_{s_t}\boldsymbol{\theta}_t)'1_{\{s_t=AA\}}|\mathcal{Y}_n, \mathcal{S}_n, \widehat{\Phi}_{\text{old}})\big]\Big/\Sigma_{t=1}^n 1_{\{s_t=AA\}}, \qquad (4)\\
\widehat{\Sigma}_{AB,\text{new}} &= \Sigma_{t=1}^n E\big[(\mathbf{y}_t - X_{s_t}\boldsymbol{\theta}_t)(\mathbf{y}_t - X_{s_t}\boldsymbol{\theta}_t)'1_{\{s_t=AB\}}|\mathcal{Y}_n, \mathcal{S}_n, \widehat{\Phi}_{\text{old}})\big]\Big/\Sigma_{t=1}^n 1_{\{s_t=AB\}},\\
\widehat{\Sigma}_{BA,\text{new}} &= \Sigma_{t=1}^n E\big[(\mathbf{y}_t - X_{s_t}\boldsymbol{\theta}_t)(\mathbf{y}_t - X_{s_t}\boldsymbol{\theta}_t)'1_{\{s_t=BA\}}|\mathcal{Y}_n, \mathcal{S}_n, \widehat{\Phi}_{\text{old}})\big]\Big/\Sigma_{t=1}^n 1_{\{s_t=BA\}},\\
\widehat{\Sigma}_{BB,\text{new}} &= \Sigma_{t=1}^n E\big[(\mathbf{y}_t - X_{s_t}\boldsymbol{\theta}_t)(\mathbf{y}_t - X_{s_t}\boldsymbol{\theta}_t)'1_{\{s_t=BB\}}|\mathcal{Y}_n, \mathcal{S}_n, \widehat{\Phi}_{\text{old}})\big]\Big/\Sigma_{t=1}^n 1_{\{s_t=BB\}}.
\end{aligned}$$

It can be shown that

$$E(\boldsymbol{\theta}_t \mathbf{1}_{\{\boldsymbol{\mu}_0 \neq \boldsymbol{\theta}_t \neq \boldsymbol{\theta}_{t-1}\}}|\mathcal{Y}_n, \mathcal{S}_n) = \sum_{t \le j \le n} \beta_{t,j,t}\boldsymbol{\mu}_{t,j},$$

$$E((\boldsymbol{\theta}_t - \widehat{\boldsymbol{\mu}})(\boldsymbol{\theta}_t - \widehat{\boldsymbol{\mu}})'\mathbf{1}_{\{\boldsymbol{\mu}_0 \neq \boldsymbol{\theta}_t \neq \boldsymbol{\theta}_{t-1}\}}|\mathcal{Y}_n, \mathcal{S}_n) = \sum_{t \le j \le n} \beta_{t,j,t}(\boldsymbol{\mu}_{t,j}\boldsymbol{\mu}'_{t,j} + V_{t,j} - 2\boldsymbol{\mu}\boldsymbol{\mu}'_{t,j} + \boldsymbol{\mu}\boldsymbol{\mu}'),$$

which can be applied to compute $\widehat{\boldsymbol{\mu}}_{\text{new}}$ and $\widehat{V}_{\text{new}}$ in (4). The iterative scheme (4) is carried out until convergence or until some prescribed upper bound on the number of iterations is reached.

To speed up the computations involved in the preceding EM algorithm, one can use the BCMIX approximations instead of the full recursions to determine $q_{i,t}, \widetilde{q}_{j,t}$, etc. Moreover, one can accelerate the EM algorithm by using a hybrid approach that combines EM with some classical optimization technique, e.g., quasi-Newton methods as in Lange (1999) [2]. Applications to array-CGH data have shown that the EM estimates of $\boldsymbol{\mu}, V, \sigma^2$ and b typically converge quite fast. This suggests switching, after these parameter estimates stabilize, from the EM algorithm to global search for the optimizing p and c, which are particularly important as they represent relative frequencies of departures from, and returns to, the baseline state. The global search in this hybrid procedure uses (2) as a function only of p and c, with the other parameter estimates fixed at the time of switch from EM.

2 BCMIX approximations

Although the Bayes method uses a recursive updating formula for the weights $q_{i,t}$ $(1 \le i \le t)$, the number of weights increases with t, resulting in rapidly increasing computational complexity and memory requirements in estimating $\boldsymbol{\theta}_t$ as t keeps increasing. A simple idea to lower the complexity is to keep only a fixed number k of weights at every stage t (which is tantamount to setting the other weights to 0). We keep the most recent m weights $q_{i,t}$ (with $t-m < i \le t$) and the largest $k-m$ of the remaining weights, where $1 \le m < k$. Specifically, the updating formula (9) in the manuscript for the weights $q_{i,t}$ is modified as follows to obtain a bounded complexity mixture (BCMIX) approximation. Let $\mathcal{K}_{t-1}$ denote the set of indices i for which $q_{i,t-1}$ is kept at stage $t-1$; thus $\mathcal{K}_{t-1} \supset \{t-1, , \cdots, t-m\}$. At stage t, define $q^*_{i,t}$ by formula (9) in the manuscript for $i \in \{t\} \cup \mathcal{K}_{t-1}$ and let i_t be the index not belonging to $\{t, t-1, \cdots, t-m+1\}$ such that

$$q^*_{i_t,t} = \min\{q^*_{i,t} : j \in \mathcal{K}_{t-1} \quad \text{and} \quad j \le t-m\}, \tag{5}$$

choosing i_t to be the one farthest from t if the minimizing set in (5) has more than one element. Define $\mathcal{K}_t = \{t\} \cup (\mathcal{K}_{t-1} - \{i_t\})$ and let

$$p_t = p^*_t \Big/ \Big(p^*_t + \sum_{j \in \{t\} \cup \mathcal{K}_{t-1}} q^*_{j,t}\Big), \tag{6}$$

$$q_{i,t} = \Big(q^*_{i,t} \Big/ \sum_{j \in \mathcal{K}_t} q^*_{j,t}\Big)\Big(\sum_{j \in \{t\} \cup \mathcal{K}_{t-1}} q^*_{j,t} \Big/ [p^*_t + \sum_{j \in \{t\} \cup \mathcal{K}_{t-1}} q^*_{j,t}]\Big), \quad i \in \mathcal{K}_t. \tag{7}$$

For the smoothing estimate $E(\boldsymbol{\theta}_t | \mathcal{Y}_n)$ and its associated posterior distribution, we can construct BCMIX approximations by combining forward and backward BCMIX filters, which have index sets $\mathcal{K}_t$ for the forward filter and $\widetilde{\mathcal{K}}_{t+1}$ for the backward filter at stage t. The BCMIX approximation

$$\alpha_t \delta_0 + \sum_{i \in \mathcal{K}_t, j \in \{t\} \cup \widetilde{\mathcal{K}}_{t+1}} \beta_{i,j,t} N(\boldsymbol{\mu}_{ij}, V_{ij})$$

to formula (7) in the manuscript is defined by

$$\alpha_t = \alpha^*_t / A_t, \qquad \beta_{i,j,t} = \beta^*_{i,j,t} / A_t, \qquad A_t = \alpha^*_t + \sum_{i \in \mathcal{K}_t, j \in \{t\} \cup \widetilde{\mathcal{K}}_{t+1}} \beta^*_{i,j,t},$$

$$\alpha^*_t = p_t[(1-p)\widetilde{p}_{t+1} + c\widetilde{q}_{t+1}]/c,$$

$$\beta^*_{i,j,t} = \begin{cases} q_{i,t}(p\widetilde{p}_{t+1} + b\widetilde{q}_{t+1})/p, & i \in \mathcal{K}_t, j = t, \\ a q_{i,t} \widetilde{q}_{j,t+1} \psi_{i,t} \psi_{t+1,j} / (p\psi\psi_{i,j}), & i \in \mathcal{K}_t, j \in \widetilde{\mathcal{K}}_{t+1}. \end{cases}$$

3 Accuracy of Genotyping

We expand here on the section "Accuracy of Estimation of Genotype States" in the main paper. Table 1 shows the number of each type of misclassification among the 42037 SNPs in HapMap sample NA06991 in the simulation data with different levels of normal cell contamination. As in the main paper we see that the number of misclassifications declines rapidly with increased normal cell contamination.

Figure 1 and Figure 2 show how the model performs in estimating the parental allele configurations at two separate normal contamination ratios (5% and 15%). We can see that as long

as the normal contamination exceeds 15%, the error in estimating parental allele configurations is very low. On the other hand, when the normal contamination is below 15%, the data values for the homozygous and heterozygous SNPs merge together in the regions containing loss of heterozygosity, and it becomes very hard to distinguish them using only the tumor data.

4 Example of Segmentation of an Affymetrix Sample

We applied PSCN to two samples analyzed through the Affymetrix platform: Chromosome 2 of TCGA glioblastoma sample 23-1027 and chromosome 17 of SW1417, a breast cancer sample. The former one does not contain non-polymorphic markers while the latter one does.

Figure 3 shows the major and minor copy number estimates of chromosome 2 of TCGA sample 23-1027, along with the sum of A and B intensities (R) and BAF. With so many data points, it is hard to visually assess the segmentation result, although the gain in total copy number at around SNP# 40000 and the split in BAF in the q-arm are clearly visible by eye. PSCN identified the gain at 40000 as a partial gain/normal event and the split in BAF as a balanced gain/loss event, which seems reasonable.

Many events are identified between SNPs 1 to 40000. For closer inspection, Figure 4 zooms in to SNPs 5001 to 10000. We see that, at the fine level, PSCN captures all of the visible splits in the BAF profile and the local shifts in the R profile, eg. the short rises in R near SNP 7500 and 9200. The many balanced gain/loss events detected in this region are visibly obvious from the BAF profile. Whether these "gain/loss regions" are inherited blocks of homozygous SNPs or somatic LOH can not be determined without a matched normal sample.

Figure 5 shows the major and minor copy number estimates for chromosome 17 of SW1417, a breast cancer sample, along with its R and BAF profiles. The visibly detectable upward shift in R at around SNP 14000 is captured by the program. The program determines that, prior to SNP 14000, the chromosome contains a mix of unbalanced gain/loss regions and a normal/loss region; with the events after SNP 14000 being mainly balanced gain/loss. Note that the program is data-adaptive in the sense that it does not give a fixed value for what a normal R should be. Instead, it uses the median of R across all of the chromosomes in a sample as a guess for the normal R. In this case, we only analyzed the data on chromosome 17. We could also argue that the events before SNP 14000 are copy neutral gain/loss and that the events after SNP 14000 are either gain/normal or unbalanced gain/loss with more in gain. Without a good estimate of the ploidy of the sample, we do not have enough information to determine whether the latter argument or the one we got from the program is closer to the truth. Estimation of ploidy in a possibly contaminated, highly differentiated sample is a difficult problem, which could be resolved by molecular cytogenetic analyses such as 24-colour karyotyping [3]. More data from the same experiment on the same sample would be helpful in resolving this issue since it would provides a better guess for normal R. Nevertheless, the program provides reasonable results with the limited information. Figure 6 zooms in to SNPs 20001 to 25000. We can see from the figure that the program captures the local trend of R at around SNP 24500.

5 Robustness to the Violation of the Gaussian Error Assumption

Figures 7 and 8 examine the adherence to Gaussianity of the errors. In testing the performance of the model, we used two simulated data sets. One is from Staaf et al. [4], which is a dilution

data set based on experimental 550k Illumina data for HapMap sample NA06991. Within this data set, one sample corresponds to normal cell contamination 100%, which does not contain any long somatic copy number aberrations, and hence can be used to analyze the noise we feed into the model. (The sample does contain very short regions of germline copy number variants, but these make up such a small fraction of the data (estimated at $< 1\%$) that they do not affect the bulk behavior of the noise.) We examined the noise of A allele intensity for BB, BA, AB, AA states through histograms and Q-Q plots. From the figures, it is clear that the true distribution deviates from multivariate Gaussian. However, as we have shown in the manuscript, the model performs quite well for this dilution data set and the simulated data set, which is obtained by adding signals to this HapMap sample NA06991 with 100% normal cell contamination. This shows that the estimation method is robust to violation of the Gaussian error assumption.

References

[1] Lai TL, Xing H, Zhang NR (2008) Stochastic segmentation models for array-based comparative genomic hybridization data analysis. Biostatistics 9: 290-307.

[2] Lange K (1999) Numerical Analysis for Statisticians. New York, Heidelberg, Berlin: Springer-Verlag.

[3] Davidson J, Gorringe K, Chin SF, Orsetti B, Besret C, et al. (2000) Molecular cytogenetic analysis of breast cancer cell lines. British Journal of Cancer 83: 1309–1317.

[4] Staaf J, Lindgren D, Vallon-Christersson J, Isaksson A, Göransson H, et al. (2008) Segmentation-based detection of allelic imbalance and loss-of-heterozygosity in cancer cells using whole genome SNP arrays. Genome Biology 9: R136+.

Normal Contamination (%)	AA $\rightarrow$ AB	BB $\rightarrow$ AB	AB $\rightarrow$ AA	AB $\rightarrow$ BB
0	893	392	1373	1418
5	735	251	1	0
10	165	63	0	0
15	42	22	0	0
20	18	13	0	0
25	11	9	0	0
30	8	6	0	0
35	20	5	0	0
40	32	14	0	0
45	54	24	0	0
50	65	28	0	0
55	104	54	0	0
60	95	43	0	0
65	88	45	0	0
70	54	28	0	0
75	61	25	0	0
80	61	31	0	0
85	61	29	0	0
90	23	16	0	0
95	18	8	0	0
100	62	22	0	0

Table 1: The count of the four types of incorrect estimation of parental allele configurations among the 42037 SNPs in HapMap sample NA06991 in the simulation data with different levels of normal cell contamination

.

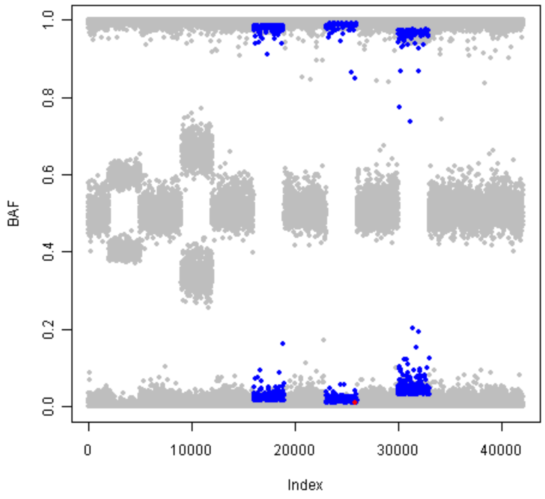

Figure 1: The BAF plot for normal contamination 5%. The SNPs with parental allele configuration correctly estimated are shown in grey. The SNPs with parental allele configuration incorrectly estimated are shown in red (AA/BB → AB) and blue (AB → AA/BB).

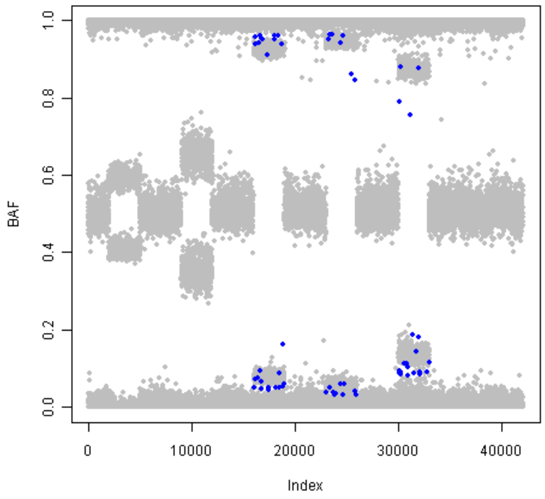

Figure 2: The BAF plot for normal contamination 15%. The SNPs with parental allele configuration correctly estimated are shown in grey. The SNPs with parental allele configuration incorrectly estimated are shown in red (AA/BB → AB) and blue (AB → AA/BB).

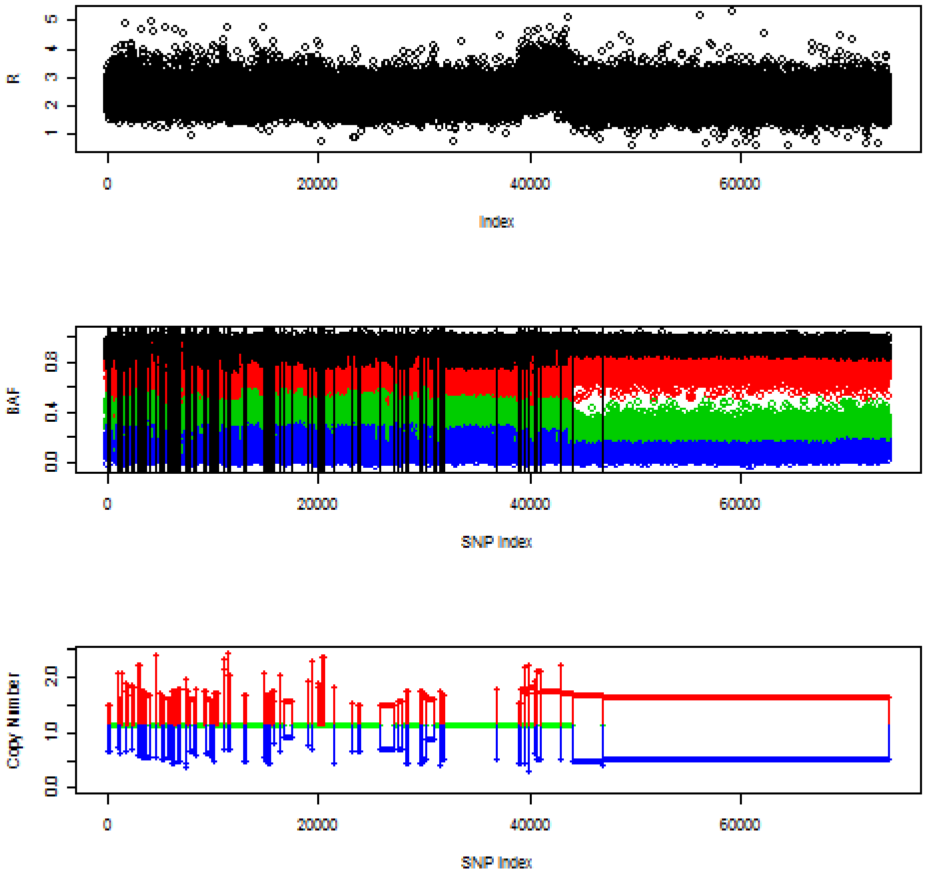

Figure 3: Plot of R, BAF, copy number estimation of Chromosome 2 of TCGA-23-1027 analyzed through the Affymetrix platform.

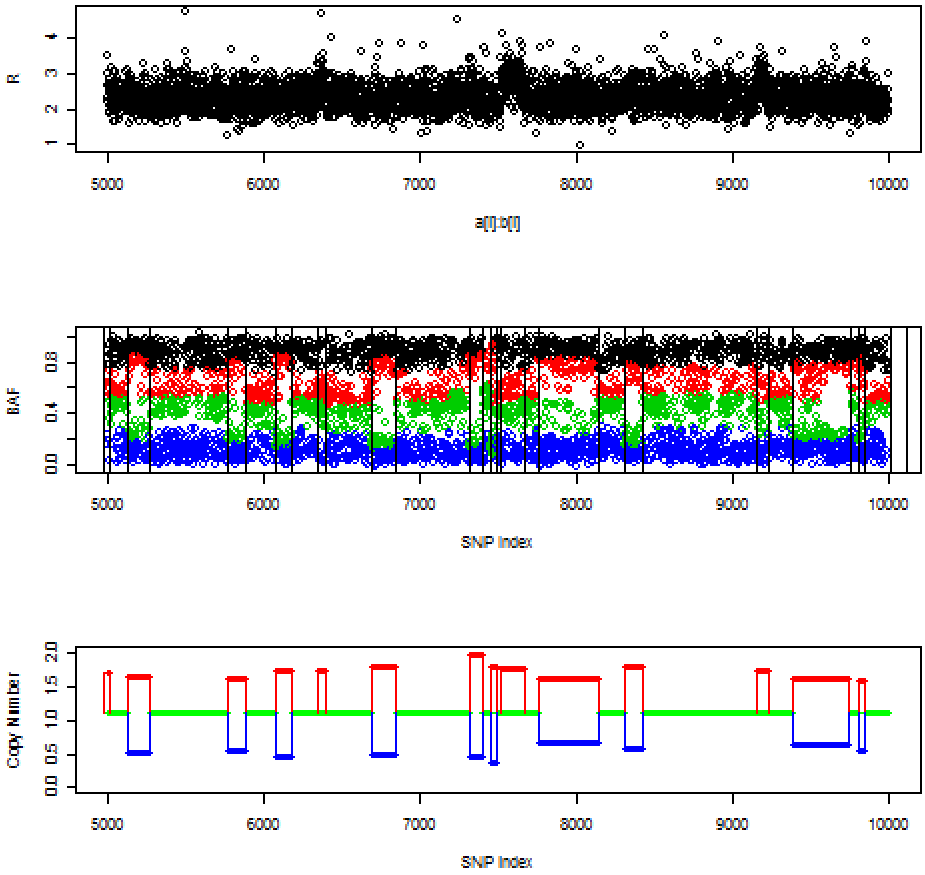

Figure 4: Plot of R, BAF, copy number estimation of Chromosome 2 of TCGA-23-1027 for SNPs 5001-10000 analyzed through the Affymetrix platform.

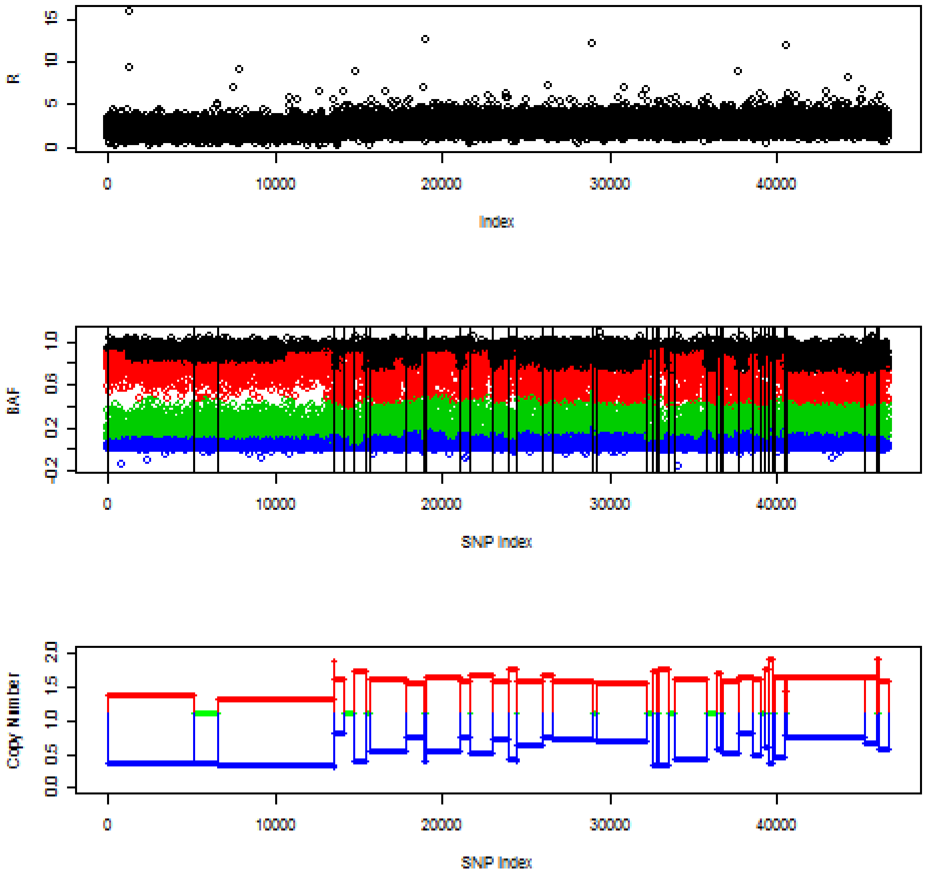

Figure 5: Plot of R, BAF, copy number estimation of Chromosome 17 of SW1417, a breast cancer sample, analyzed through the Affymetrix platform.

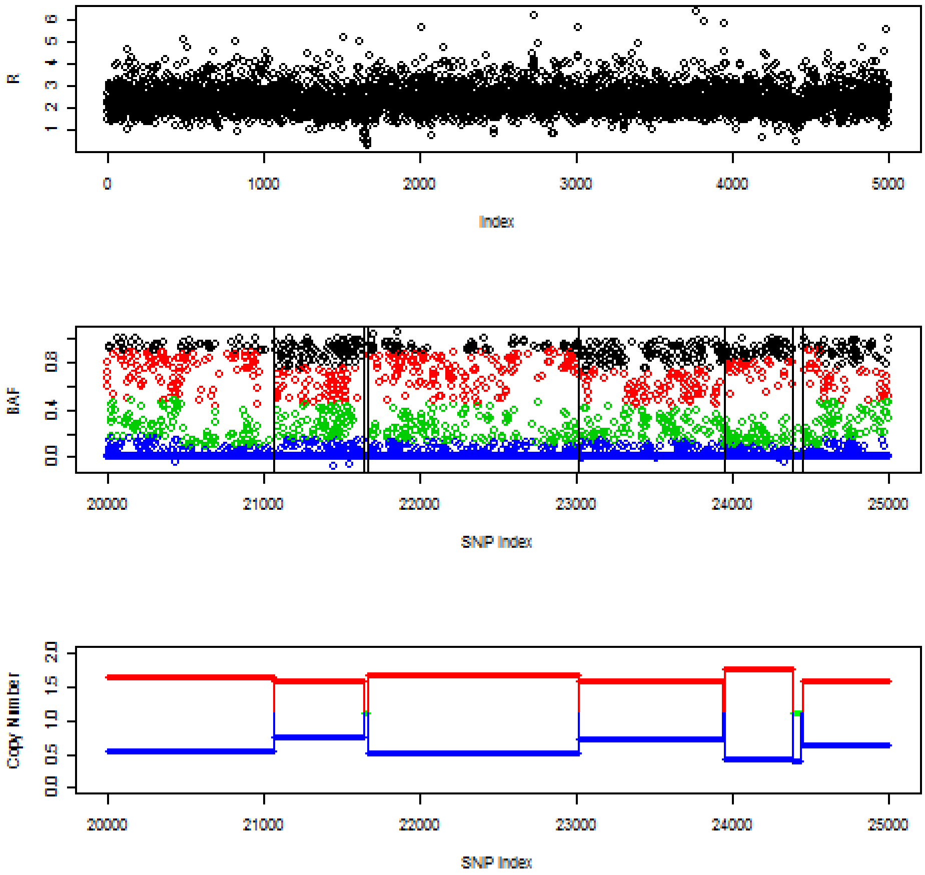

Figure 6: Plot of R, BAF, copy number estimation of Chromosome 17 of SW1417 for SNPs 20001-25000 analyzed through the Affymetrix platform.

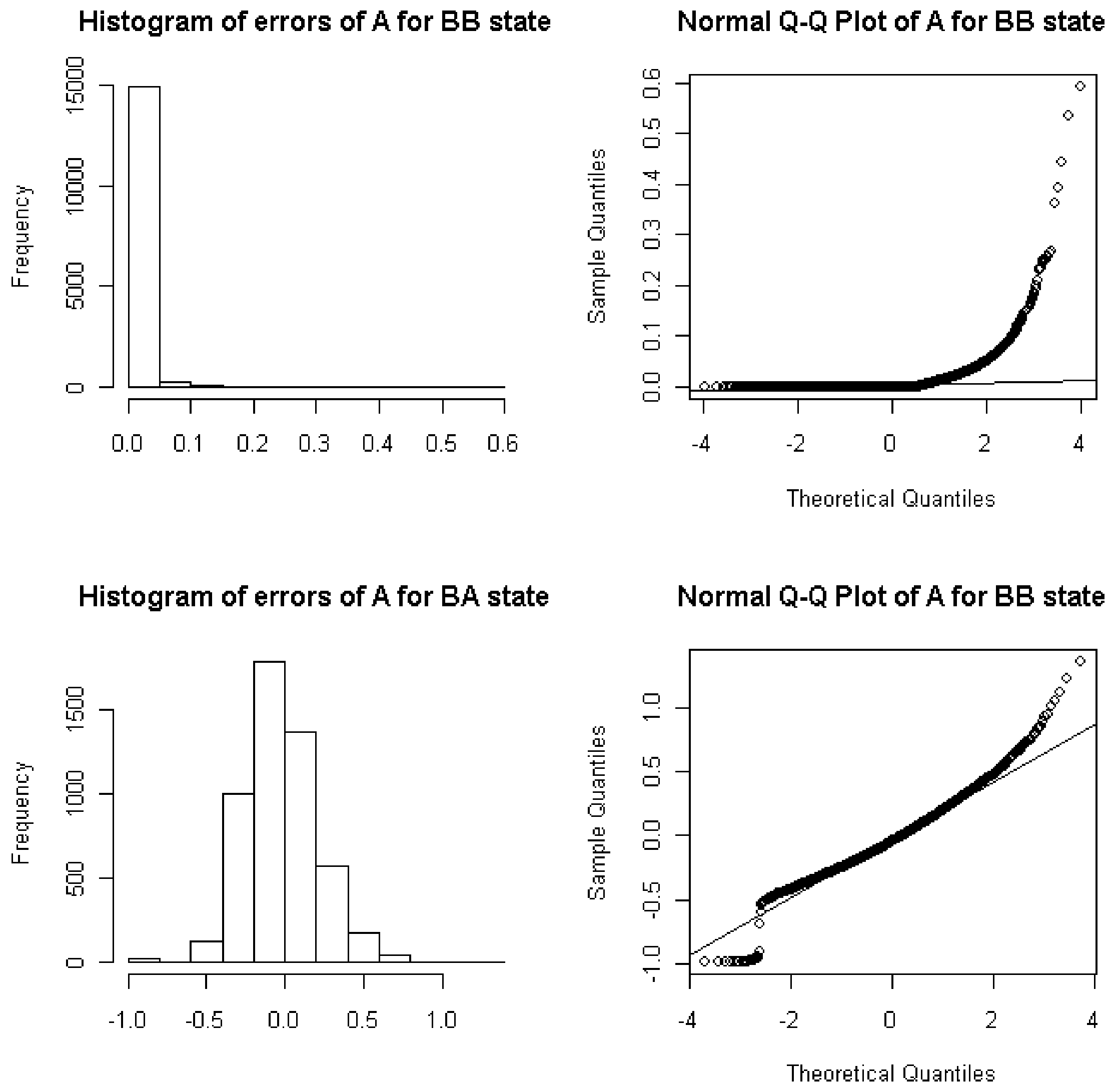

Figure 7: Histograms and Q-Q plots for errors of the fit of a chromosome from a normal cell.

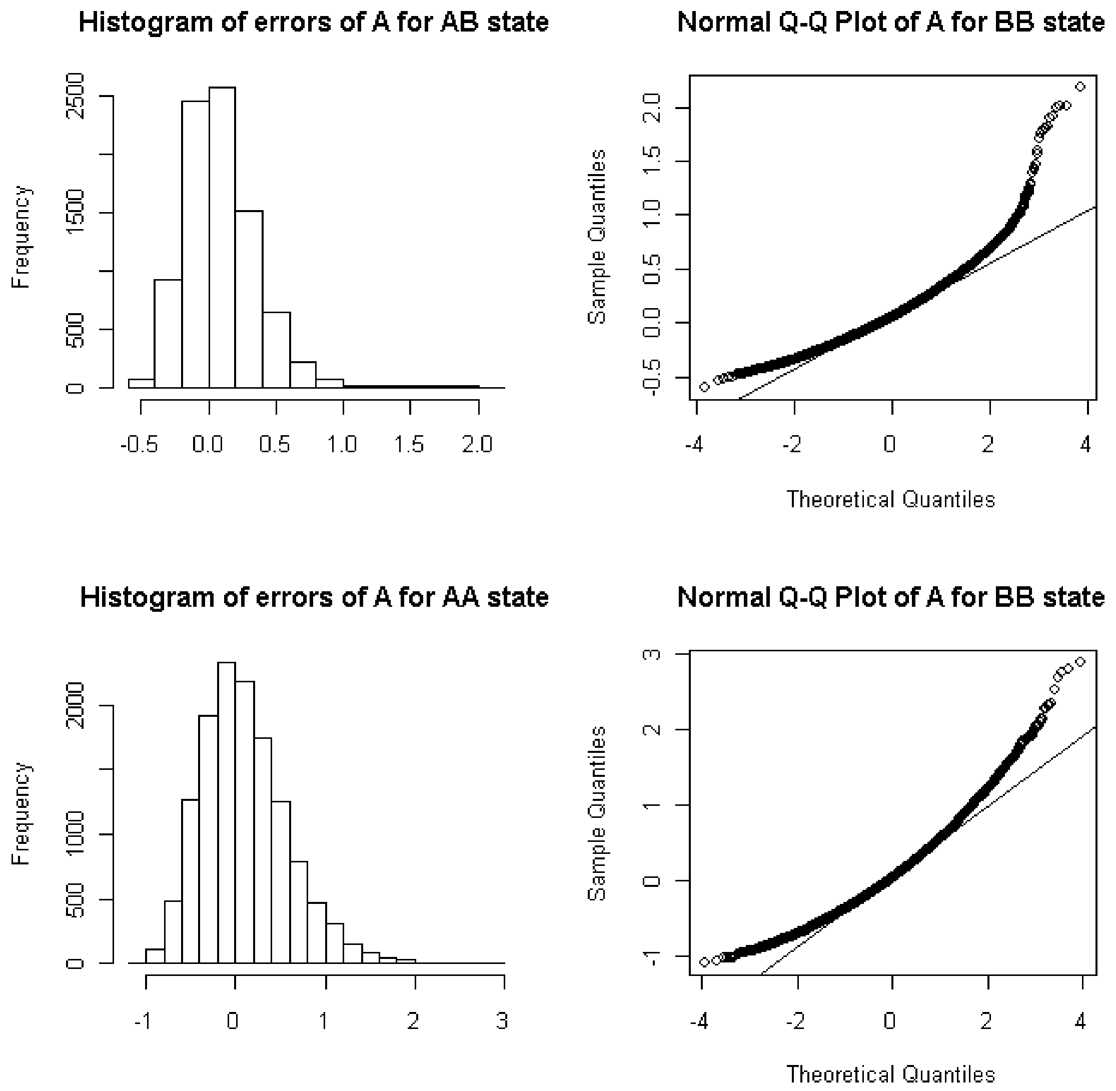

Figure 8: Histograms and Q-Q plots for errors of the fit of a chromosome from a normal cell (continued).
